# Proteome alterations during clonal isolation of established human pancreatic cancer cell lines

**DOI:** 10.1007/s00018-022-04584-9

**Published:** 2022-10-22

**Authors:** P. Bernhard, T. Feilen, M. Rogg, K. Fröhlich, M. Cosenza-Contreras, F. Hause, C. Schell, O. Schilling

**Affiliations:** 1 Institute for Surgical Pathology, Medical Center-University of Freiburg, Faculty of Medicine, University of Freiburg, Breisacher Str. 115A, 79106 Freiburg, Germany; 2 Spemann Graduate School of Biology and Medicine (SGBM), University of Freiburg, Freiburg, Germany; 3 Faculty of Biology, University of Freiburg, Freiburg, Germany; 4 Present Address: Proteomics Core Facility, Biozentrum, University of Basel, Basel, Switzerland; 5 Freiburg Institute for Advanced Studies (FRIAS), University of Freiburg, Freiburg, Germany; 6 German Cancer Consortium (DKTK), German Cancer Research Center (DKFZ), Heidelberg, Germany

**Keywords:** Limiting dilution, FACS, Single-cell Isolation, Mass spectrometry

## Abstract

Clonal isolation is an integral step of numerous workflows in genome editing and cell engineering. It comprises the isolation of a single progenitor cell from a defined cell line population with subsequent expansion to obtain a monoclonal cell population. This process is associated with transient loss of cell–cell contacts and absence of a multicellular microenvironment. Previous studies have revealed transcriptomic changes upon clonal isolation with cell line specific extent. Since transcriptome alterations are only partially reflected on the proteome level, we sought to investigate the impact of clonal isolation on the cellular proteome to a depth of > 6000 proteins in three established pancreatic cancer cell lines. We show that clonal isolation does have an impact on the cellular proteome, however, with cell line specific extent, affecting different biological processes, and also depending on the isolation method. We demonstrate a different impact of clonal isolation on mesenchymal- and epithelial-derived cell lines mainly affecting cell proliferation, metabolism, cell adhesion and cellular stress. The results bear relevance to the field of genomic editing and cell engineering and highlight the need to consider the impact of clonal isolation when interpreting data stemming from experiments that include this step.

**Supplementary Information:**

The online version contains supplementary material available at 10.1007/s00018-022-04584-9.

## Introduction

Clonal isolation describes the process of deriving a homogeneous, monoclonal cell population from a single progenitor cell, which has previously been isolated, i.e., from a parental, heterogeneous, polyclonal population. Thereby, clonal isolation can be subdivided into two successive processes, including single-cell isolation and subsequent clonal expansion of the isolated cell to a monoclonal population [1, 2].

Clonal isolation is a crucial and labor intensive step in numerous genome editing and cell engineering workflows [3, 4]. Clonal isolation is often required for the subsequent genetic and functional validation and characterization of mutations introduced by common targeted genome editing methods using zinc finger nucleases (ZFN), transcript activator-like effector nucleases (TALEN) or the CRISPR-Cas9 system [1, 4–6]. Beyond that, the isolation of hybridoma clones producing a certain monoclonal antibody or the isolation and expansion of multipotential stem cells are other applications of clonal isolation that have been described previously [7, 8].

The isolation and separation of single cells is still a technically challenging task with regard to throughput, purity and cellular viability. There are different approaches for single-cell isolation, with fluorescence-activated cell sorting (FACS) of single cells and limiting dilution cloning being the most widely used technologies [9]. While FACS requires the addition of a viability marker or stable co-expression of a fluorescent reporter and was further reported to negatively affect cell viability [10], limiting dilution cloning is suggested to induce less cellular stress and does not require sophisticated instruments or reagents, other than standard or automated pipetting tools [11, 12]. During limiting dilution, the initial cell suspension is highly diluted and plated on 96-well plates with the aim to obtain single cell-derived clones that are isolated and expanded subsequently [13, 14]. Due to the statistical nature of obtaining a single cell after dilution, this clonal isolation method is comparatively inefficient and laborious [9]. However, due to its ease of use, it is still widely applied in the field of cell engineering [15–17].

Although limiting dilution represents the gentler approach for single-cell isolation and thereby is especially recommended for the production of monoclonal cell lines, both mentioned approaches potentially induce cellular stress, due to the sudden absence of cells´ local microenvironment consisting of cellular and non-cellular components, including growth factors, metabolites or the extracellular matrix [18]. For example, the ex vivo incubation of peripheral blood cells overnight was reported to induce the expression of thousands of genes [19]. Furthermore, transcriptomic studies revealed the transcriptome-wide induction of stress-related genes in dissociated neurons as well as in tissue subpopulations of muscle stem cells upon single-cell isolation [20, 21]. However, the extent of cellular responses to single-cell isolation varies substantially depending on the cell type and the respective cell line [2].

The correlation between mRNA and protein level has been described to be only limited [22–24]. Hence, we strive to additionally evaluate and validate the above mentioned findings on proteome level. To our knowledge, this is the first study to systematically investigate the impact of clonal isolation on the cellular proteome.

## Material and methods

### Cell culture, cell harvesting and cell line authentication

The human pancreatic cancer cell lines MIA PaCa-2 (RRID:CVCL_0428, passage 8) and PANC-1 (RRID:CVCL_0480, passage 2) were cultured in Dulbecco´s modified Eagle´s medium (DMEM, high glucose, GlutaMAX™ supplement; Gibco, Thermo Fisher Scientific), while the human pancreatic cancer cell line AsPC-1 (RRID:CVCL_0152, passage 5) was cultured in RPMI-1640 medium (L-glutamine supplement; Gibco, Thermo Fisher Scientific), both supplemented with 10% Fetal bovine serum (FBS; Gibco, Thermo Fisher Scientific), 100 U/mL penicillin and 100 µg/mL streptomycin at 37 °C under a humidified atmosphere containing 5% CO_2_. To ensure comparability, cells of all conditions were first cultured in 96-well flat-bottom plates (Falcon, Thermo Fisher Scientific) before the cells of five randomly chosen wells were each transferred to individual 25 cm^2^ cell culture flasks (Greiner Bio-One) and expanded until 80–90% confluency, thereby representing five replicates of each condition.

Starting from the reference cell culture described above, cells either underwent clonal isolation to obtain single-cell colonies (described below) or were subcultured for several passages by standard cell culture techniques. For the latter, confluent 96-well plates were successively subcultured for 3 times with a cell splitting ratio of 1:10 before transferring and expanding the cells in 25 cm^2^ flasks.

For cell harvesting, cell monolayers were washed with Dulbecco’s Phosphate Buffer Saline (DPBS; Gibco, Thermo Fisher Scientific) and harvested using TrypLE™ Express Enzyme trypsin–EDTA solution (Gibco, Thermo Fisher Scientific). Cell number was determined in triplicates using 0.4% (v/v) Trypan blue staining solution (NanoEntek) and an automated cell counter (Eve Automatic Cell Counter, NanoEnTek). Cell pellets were washed with DPBS, shock-frozen in liquid nitrogen and stored at − 80 °C until further processing.

All cell lines were regularly tested for mycoplasma contaminations (Eurofins Genomics). Cell line authentication based on DNA/STR profiles (Eurofins Genomics) of all cell lines had been performed at the beginning of the experiment as well as before harvesting single-cell colonies. Inspection and documentation of confluency and cell morphology was performed by light microscopy (Leica DM IL and Leica EC3) with digital image acquisition (LAS EZ, Leica Application Suite, Version 3.1.1). For length referencing, the grid of a Neubauer counting chamber was used to calibrate the scale bar in each magnification (4 × and 10 ×).

### Fluorescence staining

For fluorescence staining, respective cell lines were seeded on 50 µg/ml fibronectin (Corning) coated 8-well chamber slides (Ibidi) at a density of 12,000 cells per well. Cells were cultured in chamber slides under standard culture conditions (as described above) for 24 h. Thereafter, cells were fixed in 4% PFA (Electron Microscopy Sciences) in DPBS (Thermo Fisher Scientific) for 20 min. After fixation, samples were 3 times washed using DPBS and permeabilized by 0.1% Triton X-100 (Sigma Aldrich) in DPBS for 3 min. Thereafter, cells were stained for F-actin using fluorophore labeled Phalloidin (Thermo Fisher Scientific) and dsDNA by Hoechst 33342 (Thermo Fisher Scientific) in a permeabilization buffer for 2 h. Finally, samples were washed 5 times in DPBS and imaged in DPBS.

### Microscopy and image analysis

An inverted Zeiss Axio Observer Z1/7 microscope (Carl Zeiss AG) equipped with an Axiocam 702 mono camera, halogen lamp, Colibri 7 illumination system and fluorescence filter sets (49 DAPI, 38 GFP, 43 HE dsRed) was used for immunofluorescence and phase imaging. For generation of phase images, cells were cultured in cell culture flasks (Greiner Bio-One GmbH) and growth medium was replaced by HBSS (Thermo Fisher Scientific) directly before imaging. Cells were imaged using a 5 × objective (N-Achroplan 5 × /0.15 Ph 1) with phase 1 polarization filter and 20 × objective (LD Plan-Neofluar 20 × /0.4 Korr Ph 2) with phase 2 polarization filter. For acquisition of immunofluorescence images 10 × (Plan-Apochromat 10 × /0.45), 20 × (Plan-Apochromat 20 × /0.8) and 40 × (Plan-Apochromat 40 × /0.95 Korr) objectives were used. Images were acquired and processed using the ZEISS ZEN 3 Software (Carl Zeiss AG). Z-stacks of 40 × fluorescence images were acquired and converted to maximum intensity projections for co-presentation of cellular compartments with different focal planes (e.g., basal membrane protrusions and cell nucleus). The QuPath v0.3.2 software [25] was used for segmentation and morphometric analysis of cell nuclei from 10 × overview images using the built-in cell segmentation tool and Hoechst 33342 staining of cell nuclei. Three independent replicates and at least 216 nuclei per cell line and replicate were analyzed. For analysis of cell morphology, 20 × images of Phalloidin stained cells were used. Cells were segmented by manually corrected thresholding of the Phalloidin fluorescence signal and morphometric parameters were calculated using Fiji ImageJ v1.52. Cell shape was expressed by calculation of cell circularity [defined as 4pi(Area/Perimeter squared)]. Three independent replicates and 100 cells per cell line and replicate were analyzed.

GraphPadPrism 6 software was used for statistical analysis and visualization of morphometric parameters. Data are expressed as mean ± s.e.m. per replicate. One-way ANOVA with Dunnett’s multiple comparisons test was used. Statistical significance was defined as *p < 0.05, **p < 0.01, ***p < 0.001 or non-significant (ns). The number of independent experiments and total amount of analyzed samples are stated in the figure legends and/or the methods section.

### Functional cell culture assays

For evaluating cellular characteristics, several functional cell culture assays were performed in 96-well format with the considered cell lines MIA PaCa-2, AsPC-1 and PANC-1. Cell proliferation was tested using the colorimetric BrdU-incorporation ELISA-assay (Roche), which was performed according to manufacturer´s instructions with 5000 cells/well and 48 h incubation time for initial seeding and 4 h incubation time for BrdU labelling. Metabolic activity was evaluated using the MTT-based colorimetric, non-radioactive CellTiter 96^®^ assay (Promega) according to manufacturer’s instructions with 5000 cells/well and 48 h incubation time for initial seeding. ECM cell adhesion was determined using the colorimetric ECM Cell Adhesion Array Kit (Merck Millipore) according to manufacturer´s instructions with 150,000 cells/well in serum-free medium (DMEM or RPMI-1640) and 2 h incubation for initial adhesion. For the analysis of all assays, the mean blank value (negative control) was subtracted from all other values before further analysis. For all assays, colorimetric readout was obtained using a Tecan Spark 10 M microplate reader.

GraphPadPrism 6 software was used for statistical analysis of colorimetric readouts. Data are expressed as mean ± standard deviation per condition. One-way ANOVA with Tukey’s or Dunnett’s multiple comparisons test was used and is stated in the figure legends. Statistical significance was defined as *p < 0.05, **p < 0.01, ***p < 0.001, ****p < 0.001 or non-significant (ns). The number of independent experiments and total amount of analyzed samples are stated in the figure legends.

### Clonal isolation by limiting dilution cloning or FACS

The isolation of single-cell colonies was performed by limiting dilution as previously described [2]. In short, cell monolayers (80–90% confluency) were detached using TrypLE™ Express Enzyme trypsin–EDTA solution (Gibco, Thermo Fisher Scientific) before determining the cell number in triplicates using 0.4% (v/v) Trypan blue staining solution (NanoEntek) and an automated cell counter (Eve Automatic Cell Counter, NanoEnTek). Subsequently, cells were serially diluted in DMEM to a final concentration of 0.5 cells per 100 µL before plating two 96-well flat-bottom plates (Falcon, Thermo Fisher Scientific) with 100 µL per well. Cells were cultured like described above and regularly inspected for single-cell colonies (rounded colonies radiating from a central point). Cells of respective wells were cultured until 80–90% confluency, then transferred and expanded to 25 cm^2^ flasks before harvesting them like described above. For an additional round of clonal isolation via limiting dilution, the whole process was repeated with the already obtained single-cell colony.

For single-cell sorting via FACS, cells were detached and counted like described above before adjusting cell number to 3 × 10^7^ cells/mL in the respective medium supplemented with a final concentration of 1 µg/mL propidium iodide (PI, Sigma–Aldrich) as viability marker. FACS-assisted single-cell sorting for PI-negative cells into 96-well tissue culture plates was kindly performed by the Lighthouse Core Facility (Center for Translational Cell Research, University Medical Center Freiburg) using a CytoFlex SRT cell sorter (Beckmann Coulter) operated at 15 psi with a 100 µm nozzle. Subsequently, cells were cultured and inspected like described above.

### Protein extraction and proteomic sample preparation

For each cell line, respective cell pellets were resuspended in DPBS and equalized cell numbers were incubated with detergent-containing protein extraction buffer (0.1 M HEPES pH 7.5, 0.1% (v/v) SDS, 0.57 mM PMSF, 10 mM EDTA). Samples were heated for 10 min at 95 °C followed by ultrasonication (Bioruptor, 10 cycles, 45/15 s on/off time, high intensity) and centrifugation (500 g, 5 min), thereupon only using the clear supernatant.

For mass spectrometry (MS) sample preparation, protein concentration of each supernatant was determined using BCA (Thermo Fisher Scientific, Waltham, USA) before subjecting equalized protein amounts of each cell line (100–130 µg) to in-solution tryptic digestion. In short, cystine reduction was performed using 5 mM dithiothreitol (30 min, 37 °C) and subsequent alkylation using 15 mM iodoacetamide (30 min, room temperature, in the dark). Protein enrichment and SDS removal was performed using the previously published sp3-bead protocol [26]. Protein-coupled beads were resuspended in 0.1 M HEPES pH 8.0 containing 0.1% (v/v) of an acid-labile surfactant (sodium 3-[(2-methyl-2-undecyl-1,3-dioxolan-4-yl)methoxy]-1-propanesulfonate). On-bead proteolytic digestion was performed by adding Lysyl Endopeptidase (LysC, Wako Chemicals) in a protease:protein ratio of 1:50 (w/w) and incubating for 2 h at 42 °C, before adding trypsin (Promega) in a protease:protein ratio of 1:50 (w/w) and further incubate samples for 17 h at 37 °C. Digestion was stopped by acidification (2% (v/v) TFA), before incubating (37 °C, 30 min) and centrifuging (20,000*g*, 10 min) the samples to precipitate acid-labile surfactant. Clear supernatant was used for peptide desalting using iST C18 mixed phase cartridges (PreOmics, Martinsried, Germany) according to manufacturer´s instructions. After determining the peptide concentration via BCA (Thermo Fisher Scientific), eluates were vacuum dried and stored at − 80 °C until peptide labelling.

For peptide labelling, all 15 samples per cell line were treated as one experiment and were labelled with one set of TMTpro-16plex reagents, while leaving one channel empty [27, 28]. Therefore, a consistent peptide amount of 25 µg per sample was resuspended in 40 µL of 0.1 M HEPES pH 8.0 before adding different amounts of unlabelled iRTs to each sample as internal labelling control. Subsequently, each sample was mixed with 0.2 mg of one specific TMTpro-reagent (dissolved in 10 µL DMSO) and was incubated overnight at room temperature under constant agitation (500 rpm). TMT-labelling was quenched by heating to 80 °C for 15 min before pooling all samples to one TMT-mixture per cell line and diluting DMSO to less than 10% (v/v) with 10 mM Ammonium formate. After incubating the pooled sample for 20 min at room temperature and subsequent centrifugation (20,000*g*, 10 min), supernatant corresponding to 80 µg of labelled peptides was fractionated by offline high pH reversed phase chromatography as described previously [29], resulting in 12 fractions per cell line, which were vacuum dried and stored at − 80 °C until measurement.

### TMTpro-16plex HEK-E.Coli Benchmark Dataset

*E.Coli* DH5α bacteria as well as HEK cells were separately lysed in 100 mM HEPES pH 7.5 supplemented with 0.1% (v/v) SDS, 0.57 mM PMSF and 10 mM EDTA, heated for 10 min at 95 °C followed by ultrasonication (Bioruptor, 20 cycles, 45/15 s on/off time, high intensity) and centrifugation (13,000*g*, 5 min), thereupon only using the clear supernatant. Protein concentration of each supernatant was determined using BCA (Thermo Fisher Scientific, Waltham, USA) before subjecting 1 mg of each lysate to individual in-solution tryptic digestion as described above. Peptide desalting was performed using Sep-Pak C18 Plus Short Cartridge (Waters, Milford, USA) according to manufacturer´s instructions. After determining the peptide concentration via BCA (Thermo Fisher Scientific, Waltham, USA), 25 µg HEK eluate were mixed with 0.5 µg, 1.5 µg, 4.0 µg or without *E. coli* eluate to obtain peptide mass ratios of *E.coli*:HEK 1:50, 1:17, 1:6 and “HEK only”, respectively. For each mass ratio, four replicates were prepared resulting in 16 samples in total. Isobaric labelling with TMTpro-16plex and subsequent offline high pH fractionation was performed like described above before resulting fractions were vacuum dried and stored at − 80 °C until measurement.

### Mass spectrometry measurement

For MS measurement, vacuum dried peptides were resolubilized in 0.1% (v/v) formic acid to a final concentration of 0.2 µg/µL, sonicated for 5 min and centrifuged at 20,000*g* for 10 min before transferring the supernatant to the measurement tube. 800 ng of each sample, together with 100 fmol of unlabelled indexed retention time (iRT) peptides, were analysed using a nanoflow liquid chromatography (LC) system, Easy-nLC 1000 (Thermo Fisher Scientific, Waltham, USA) equipped with a trapping column (50 cm µPac™ trapping column, PharmaFluidics, Ghent, Belgium) and an analytical column (200 cm µPac™ analytical column, PharmaFluidics, Ghent, Belgium) tempered to 45 °C. Samples were trapped at 200 bars with 100% buffer A (0.1% v/v formic acid) and separated using a dynamic flow rate of 350–700 nL/min. A multistep gradient of 8% to 55% buffer B (80% v/v acetonitrile, 0.1% v/v formic acid) in buffer A was used for separation, followed by washing (100% B) and reconditioning of the column to 8% B (Sup. Table 1 for detailed gradient overview).

For the analysis of MIA PaCa-2 samples, 500 ng of each sample, together with 100 fmol of unlabelled indexed retention time (iRT) peptides, were subjected to MS measurement. The Easy-nLC 1000 was equipped with a trapping column (Fused Silica Capillary; 5 cm length, 100 μm inner diameter, VICI Jour, Schenkon, Switzerland) and an analytical column (Self-Pack PicoFrit Column; 35 cm length, 75 μm inner diameter, New Objective, Woburn, USA) both in-house packed [30] with C18 particles (Dr. Maisch, ReproSil-Pur 120 C18-AQ; 1.9 μm C18 particle size, 120 Å pore size). Samples were trapped at 400 bar with 100% buffer A (0.1% v/v formic acid) and separated using the reverse phase analytical column tempered to 60 °C at a flow rate of 400 nL/min. A multistep gradient of 11% to 70% buffer B (80% v/v acetonitrile, 0.1% v/v formic acid) in buffer A was used for separation, followed by washing (100% B) the column (Sup. Table 2 for detailed gradient overview).

For all investigated cell lines, the Easy-nLC 1000 system was coupled online to a Q-Exactive plus (Thermo Fisher Scientific, Waltham, USA) mass spectrometer via a Nanospray Flex Ionsource (Thermo Fisher Scientific, Waltham, USA) with an applied voltage of 2.1 kV for electrospray ionization. Due to the respective column setup, the analytical column was coupled to a pulled, uncoated ESI emitter (10 μm tip inner diameter, 20 µm inner diameter, 7 cm length, CoAnn Technologies, Richland, USA) via a µPac™ Flex iON Connect ESI–MS interface (PharmaFluidics, Ghent, Belgium) for the samples of AsPC-1 and PANC-1, whereas for MIA PaCa-2 the analytical PicoFrit column contains an integrated uncoated pre-cut emitter (Silica Tip, 10 μm tip inner diameter, New Objective, Woburn, USA). The mass spectrometer was operated in data dependent acquisition (DDA) mode and each MS scan was followed by a maximum of 10 MS/MS scans (Top10 method). The mass range from 300 to 2000 *m*/*z* (mass-to-charge ratio) was analysed with a dynamic exclusion time of 35 s. MS resolution was set to 70,000, automatic gain control (AGC) to 3e6 and maximum injection time was set to 50 ms. Upon HCD-fragmentation, MS/MS resolution was set to 35,000, AGC to 1e6 and maximum injection time to 110 ms.

For targeted parallel reaction monitoring (PRM) analysis, the unlabeled samples were resolubilized and injected like described above using the same instrumental setup. However, the mass spectrometer was operated in unscheduled parallel reaction monitoring (PRM) acquisition mode. Therefore, MS2 scans (1 µscan) of doubly-charged precursor ions were performed with an isolation window size of 1.2 *m*/*z*, MS2 resolution was set to 35,000, AGC to 3e6 and maximum injection time was set to 150 ms using stepped NCE of 25 and 30 for fragmentation. Samples were measured in randomized sample order.

### Proteomic data analysis

For proteomic data analysis of DDA data, each cell line was analysed individually as an independent experiment. MaxQuant (V2.0.1.0) software was used for data analysis [31]. Peptide identification was performed using the Andromeda search engine [32] with a human proteome database containing reviewed Uniprot sequences without isoforms downloaded from Uniprot on 14th June 2021 (20,856 entries). Decoys for the database search were generated in MaxQuant using the revert function. The precursor mass tolerance for the initial search was 20 ppm and for the main search 4.5 ppm, whereas the fragment mass tolerance was 20 ppm. Tryptic cleavage specificity (Trp/P) with 2 missed cleavages was applied while setting a minimal peptide length of seven amino acids. Carbamidomethyl at cysteines was the only fixed modification, whereas setting methionine oxidation and protein N-terminal acetylation as variable modifications allowing a maximum of 5 modifications per peptide. Reporter ion MS2 with TMTpro16-plex was chosen with a precursor intensity fraction (PIF) of at least 0.5. The false discovery rate (FDR) for peptides and proteins was set to 0.01. Match-between-Run was enabled.

The MaxQuant output was further processed in R (V 4.1.0) with RStudio (V 2021.09.1) as an integrated development environment. After filtering for unique peptides, protein summarization, log2-transformation and median centering was performed using the MSstatsTMT package (V 2.0.1) with default settings [33]. After validating correct TMT-channel assignment by comparing iRT-intensities as internal labelling control, Biognosys iRT-peptide entries were removed from the protein list. Partial least squares discriminant analysis (PLS-DA) and principal component analysis (PCA) were performed using the mixOmics package (V 6.16.3) with default settings except for defining three components [34]. Cluster of proteins that are consistently co-expressed were identified using the Clust algorithm (V 1.10.10), which assigns each identified protein to a certain abundance-course with a confidence interval of 95% [35]. Clust algorithm was executed in Python (V 3.8.9) using *Z*-score normalization prior to *k*-means clustering with seed number *k* = 6 and tightness weight *t* = 1.0. Differential expression analysis was performed with a multigroup limma approach using the limma package (V 3.48.3) for pairwise statistical testing [36]. Proteins, which revealed a significant abundance change (adjusted *p* value ≤ 0.05, Benjamini–Hochberg correction) together with an absolute log2 fold change ≥ 0.13 (corresponds to ~ 10% fold change) were considered as differentially expressed proteins. Gene Ontology annotation (Biological Process) of differentially expressed proteins was performed using the enrichGO-function of the clusterProfiler package (V 4.0.5) with default settings and using the entirety of identified proteins as background [37] or using STRING site version 11.5.

TMT labelling efficiency was checked with an additional MaxQuant analysis using the same parameters as described above, except for setting the standard label-free LC–MS type with N-terminal and Lysin TMTpro-16plex-adducts as additional variable modifications. In R, contaminant and reverse peptide hits were removed before the resulting peptides were filtered for tryptic peptides containing a C-terminal arginine with zero missed cleavages. For this peptide subset, N-terminal labelling efficiency ratio was calculated based on the intensity sum of peptides, containing an N-terminal TMTpro-16plex-adduct, compared to the overall intensity sum within this subset.

For proteomic data analysis of PRM data, the open-source software tool Skyline (version 19.1) was used [38]. All integrated peaks were manually inspected to ensure correct peak detection and integration. The resulting peak areas of respective proteins were subsequently normalized to the protein folylpolyglutamate synthase (Uniprot-ID: Q05932), which revealed consistent abundance over all samples due to limmaTOST analysis using the ezlimma package (V 0.2.5.9000). For statistical evaluation, a two-sample t-test was performed using Benjamini–Hochberg for multiple testing correction. Resulting fold changes and corresponding 95% confidence intervals are visualized as bar plots.

## Results and discussion

### Clonal isolation and standard cell culture do not lead to apparent morphological changes

**Fig. 1:**
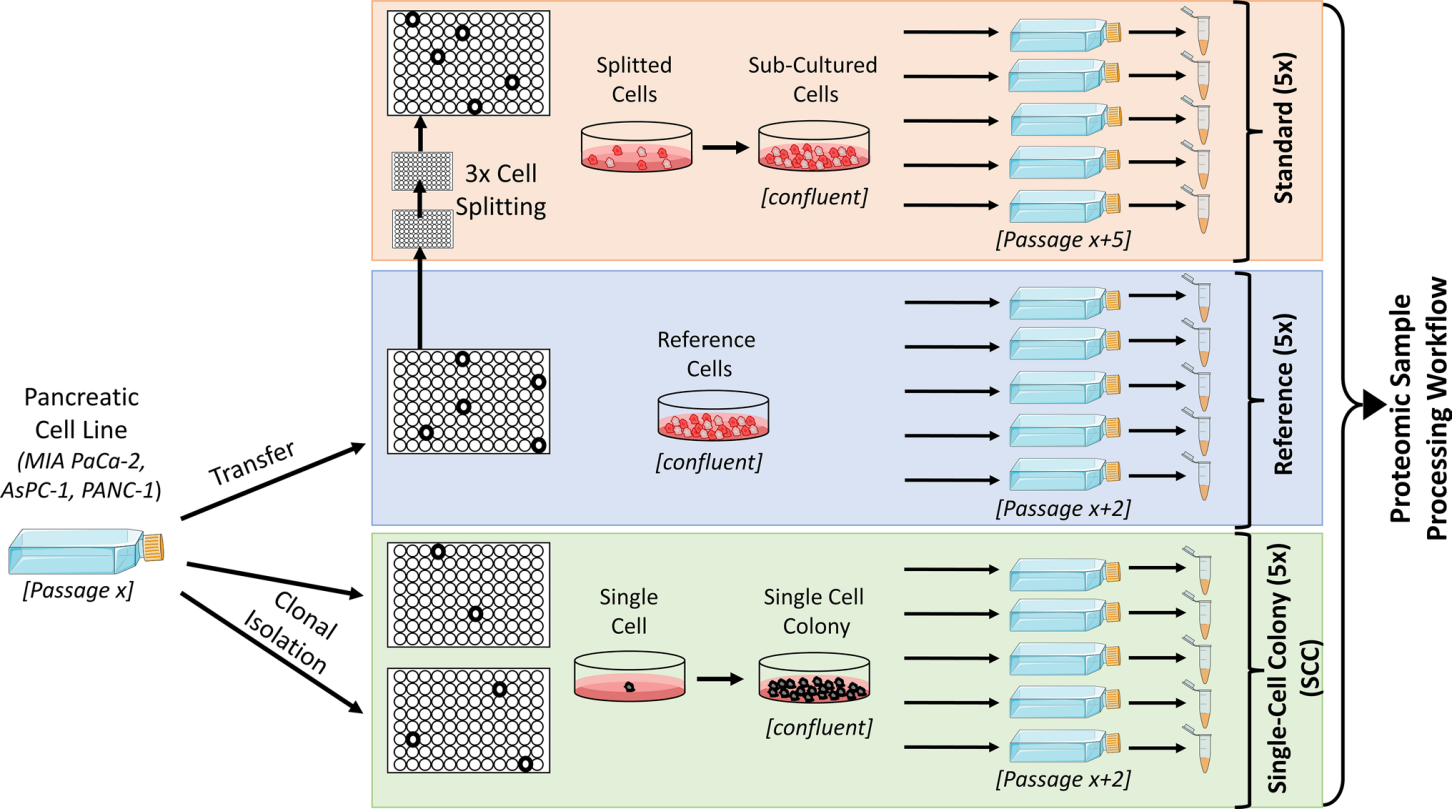
Schematic Cell Culture Workflow. The initial cell culture of human pancreatic cell lines MIA PaCa-2, AsPC-1 and PANC-1 was transferred to 96-well plates (Reference, blue). Upon confluency, cells were expanded in 25 cm^2^ flasks and harvested as cell pellets for further proteomic analysis. In parallel, initial cells underwent clonal isolation through limiting dilution to obtain single cells and were also plated on two 96-well plates. Emerging single-cell colonies were also cultured until confluency, expanded in 25 cm^2^ flasks and harvested as cell pellets (Single-Cell Colony, SCC, green). To account for an increased number of cell divisions, reference cells were further subcultured for three times according to standard cell culture techniques before expanding and harvesting as described (Standard, orange)

Starting from an initial cell culture as a reference, three human pancreatic cancer cell lines (MIA PaCa-2, AsPC-1 and PANC-1) were either maintained by standard cell culture procedures or underwent clonal isolation to obtain single-cell colonies (Fig. 1). Emerging single cells were again expanded upon confluency before harvesting them as cell pellets. To account and compensate for the increased number of cell divisions from a single cell until confluency is reached again, the cells maintained by standard cell culture were subcultured three times before harvesting them. For each condition (reference, single-cell colony and standard cell culture), five replicates were expanded and harvested individually to obtain fifteen samples in total per cell line for subsequent proteomic sample processing. The five replicates per condition are important to account for the potential clonal variability within each condition.

**Fig. 2:**
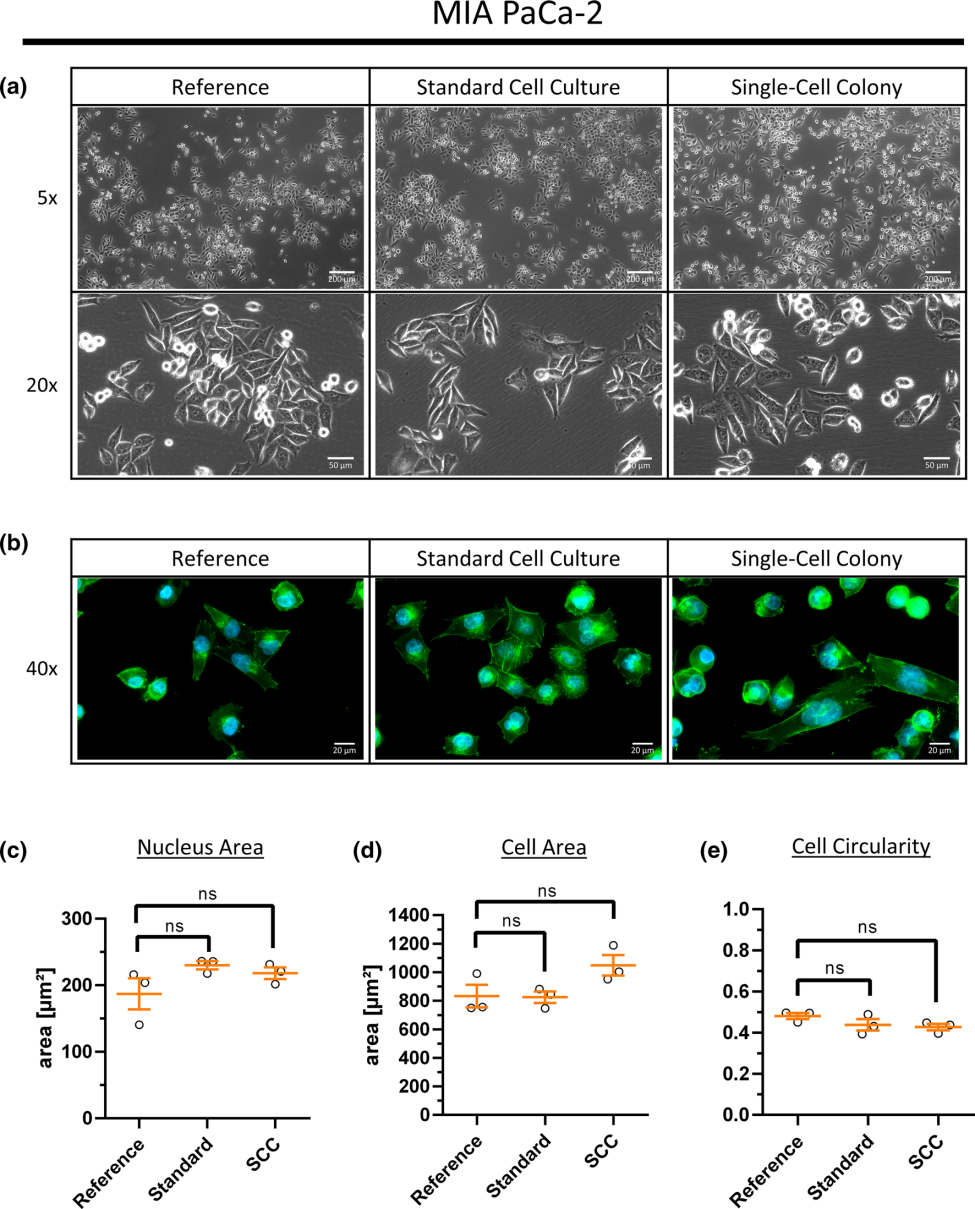
Cell Morphology before and after Standard Cell Culture or Clonal Isolation of human pancreatic MIA PaCa-2 cell line. Human pancreatic MIA PaCa-2 reference cells either undergoing standard cell culture or clonal isolation via limiting dilution to obtain single-cell colonies. **a** Cells were cultured in cell culture flasks and imaged via phase contrast microscopy (5 × and 20 × magnification). **b** Cells were fluorescence stained for F-actin by fluorophore labelled Phalloidin (green) and dsDNA by Hoechst 33342 (blue). Maximum intensity projection of 40 × *z*-stack images is shown. **c**–**e** Morphometric parameters of individual cells were analysed based on Phalloidin and Hoechst 33342 fluorescence staining. Three independent replicates and at least 329 nuclei (**c**) or 100 cells (**d**, **e**) per condition and replicate were analysed. Scatter plot dots represent mean values per replicate (error bars show mean and S.E.M., ns—non significant)

24 h after clonal isolation of MIA PaCa-2 and AsPC-1, an accumulation of four to five cells was first discovered, which proliferated over time and led to a spherical colony after 20 days, suggesting this colony to be a single-cell derived colony (Sup. Fig. 1a, b). For PANC-1, the first accumulation of cells was spotted after 72 h but also led to a spherical colony after 10 days indicating a successful single-cell isolation (Sup. Fig. 1c). During cultivation, cells were regularly inspected and documented to check for potential alterations between the three conditions. For MIA PaCa-2, no visible differences in growing behavior or morphology could be detected—neither after standard cell culture nor upon clonal isolation (Fig. 2). This suggests that clonal isolation does not completely alter overall cellular characteristics for the considered cell lines, which is supported by a successful cell line authentication before and after clonal isolation (cell authentication certificates are available online in the MassIVE repository). Similar effects could be observed for the cell lines AsPC-1 and PANC-1 (Sup. Figs. 2, 3, respectively) albeit morphometric analysis of AsPC-1 showing a significantly increased cell circularity upon clonal isolation (Sup. Fig. 2e) and PANC-1 revealing a significantly reduced cell area upon standard cell culture and clonal isolation (Sup. Fig. 3d). Both morphometric parameters suggest a reduced surface attachment and less cell stretching of the respective cells. However, for other cell cultures it has been reported that cellular morphology and growing behavior have been changed more drastically upon clonal isolation, although it was not interpreted as a result of single-cell isolation [15].

### Proteomic workflow provides comprehensive proteomic datasets

**Fig. 3:**
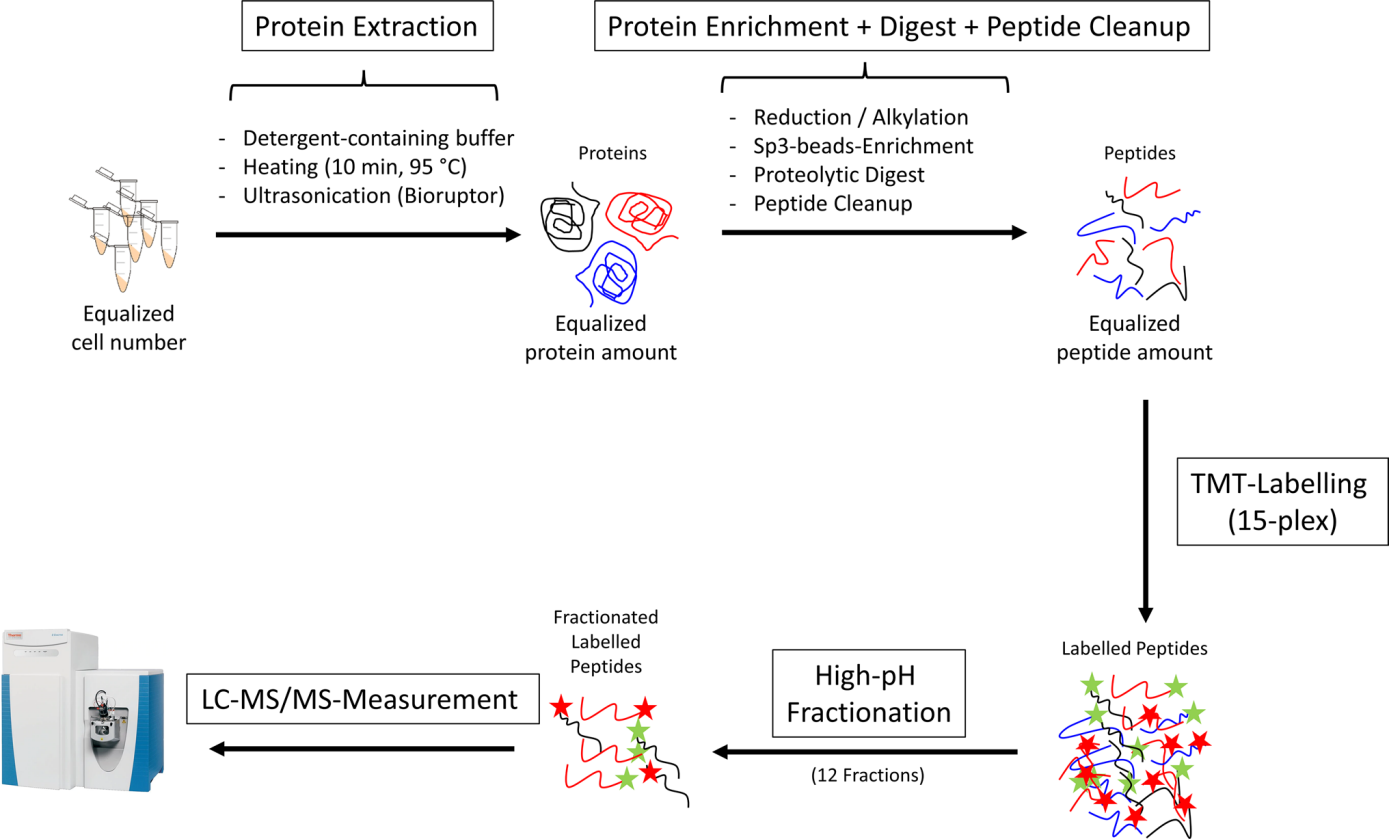
Schematic Proteomic Sample Preparation Workflow. Starting from harvested cell pellets, equalized cell numbers were incubated with detergent-containing buffer followed by heating and ultrasonication to achieve protein extraction. Extracted proteins underwent reduction, alkylation, enrichment via sp3-beads, proteolytic digestion with LysC and trypsin as well as cleanup of resulting peptides. After isobaric labelling with tandem mass tag (TMTpro-16plex; leaving one channel empty), all samples of one cell line were pooled and offline fractionated using high-pH reversed phase chromatography. Resulting 12 fractions were subjected to liquid chromatography–tandem mass spectrometry (LC–MS/MS) operated in data-dependent acquisition mode (DDA)

For proteomic sample preparation, each cell line was processed individually. Thus, 15 harvested cell pellets per cell line, including 5 replicates for each condition, were subjected to the proteomic sample preparation workflow (Fig. 3). After protein extraction, protein enrichment, proteolytic digest and isobaric peptide labelling with tandem mass tags (TMTpro-16plex; leaving one channel empty), all samples of one cell line were pooled and subjected to offline high-pH fractionation to reduce sample complexity and improve peptide and protein identifications. The resulting 12 fractions were subsequently analysed via liquid chromatography tandem-mass spectrometry (LC–MS/MS) operated in data-dependent acquisition mode (DDA), resulting in the identification and quantification of 8526–11,281 peptides and 3307–3682 proteins per fraction (Sup. Fig. 4a) and a total of 6413, 6346 and 6303 unique proteins for the cell lines MIA PaCa-2, AsPC-1 and PANC-1, respectively. The labelling efficiency of isobaric TMT-labelling on peptide-level is close to 100% for all cell lines, emphasizing an efficient labelling protocol (Sup. Fig. 4b).

### 16plex tandem mass tag pro peptide labelling is applicable for quantitative proteomics using a hybrid quadrupole–orbitrap mass spectrometer and MS2-level mass spectrometry

The concept of isobaric labelling with tandem mass tags (TMT) is widely used in the proteomic field to enable multiplex relative quantification [39–41]. Recently, 16-plex TMTpro reagents were introduced [27, 28]. To corroborate the applicability of the TMTpro labelling approach for quantitative proteomics with our instrumental setting, we performed a TMTpro-16plex benchmark analysis. Interspecies titration series are a commonly used approach to corroborate the applicability of quantitative proteomics strategies [42–45]. Therefore, we made use of this multi-species approach and analysed a benchmark dataset consisting of 16 TMT-labelled samples representing HEK-*E.coli* peptide mixtures of four defined ratios (Sup. Fig. 5a). Like our biological samples, all labelled benchmark samples were pooled and fractionated in 12 fractions to reduce sample complexity. The data revealed a TMT-labelling efficiency of 99.8% and enable the identification and quantification of 7483 unique proteins in total, consisting of 6959 unique human proteins and 524 unique *E. coli* proteins (data not shown). Resulting protein intensities showed the expected increase for *E. coli* proteins and the expected consistency for human proteins across the mixing ratios (Sup. Fig. 5b). Also the log2 fold change distribution after pairwise differential expression analysis revealed the expected centering of human protein log2 fold changes around 0, while *E. coli* proteins showed positive median log2 fold changes due to increasing amounts of *E. coli* peptides within each comparison (Sup. Fig. 5c). However, the absolute values of median fold change ratios are consistently lower than the expected values and show a reduction by a factor of 1.6–3.2. This probably represents a commonly observed behaviour of TMT and other isobaric labelling experiments called ratio compression leading to systematic underestimation of fold changes [46–48]. Compared to the literature, the herein observed compression factors are comparable with other studies using a similar experimental and instrumental setup, describing compression factors between 2.0 and 3.6 [49–51]. Moreover, despite the observed ratio compression, TMT-based quantification has been previously shown to provide high precision and thereby allow for clear discrimination between differential and constant protein abundances, with an even higher statistical significance than the widely applied Label-free Quantification (LFQ) method [49]. This validates the applicability of TMTpro-labelling with our instrumental setting and emphasizes the reliability of our acquired data for protein quantification. However, it should be noted that the described benchmark dataset only tested protein quantification for fold changes bigger than 2.7 while fold changes in biological TMT-datasets could be considerably smaller. The focus on elevated quantitative alterations in the benchmark dataset is in line with common practice in the field of proteomics [42–45, 49].

### *Global overview of proteomic profiles *via* partial least square discriminant analysis*

**Fig. 4:**
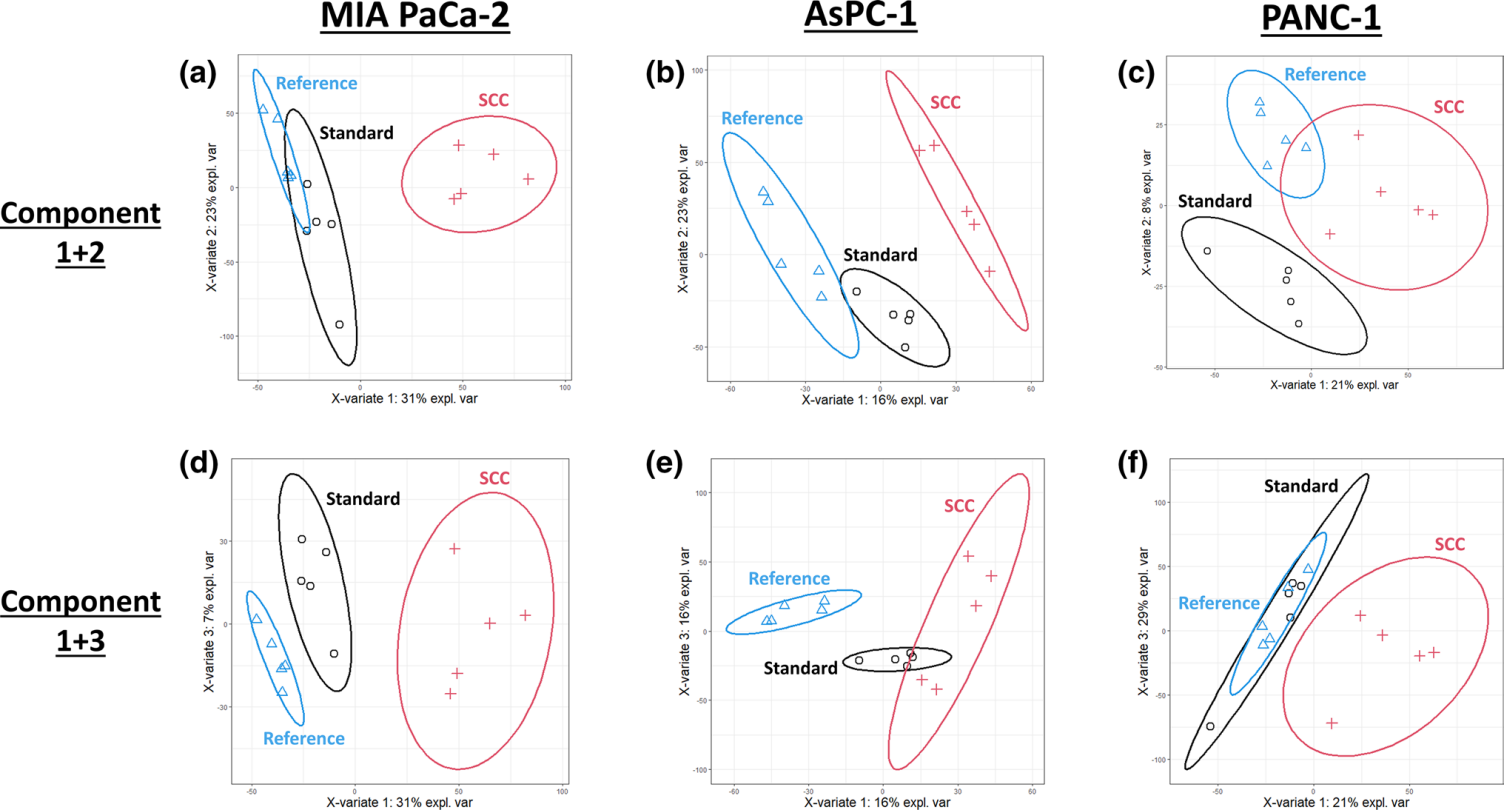
Partial Least Squares Discriminant Analysis (PLSDA). Protein profiles of each sample together with the corresponding condition annotation (Reference, Standard Cell Culture, Single-Cell Colony (SCC)) were submitted to PLSDA analysis. For the analysis, either components 1 and 2 (**a**–**c**) or components 1 and 3 (**d**–**f**) were considered, where *x*- and *y*-axis represent the percentage of explained variance of the respective component. Ellipses represent 95% confidence intervals

To explore the global effects of clonal isolation on the overall cell line proteome, the obtained proteomic data was used to perform a partial least squares discriminant analysis (PLSDA). Figure 4 shows the result of the PLSDA for each cell line, when considering components 1 and 2 or components 1 and 3.

For MIA PaCa-2, it is evident that the proteome profile of single-cell colonies (SCC) completely segregates from the reference and the standard cell culture profile, suggesting that clonal isolation does have a noticeable impact on the cell line proteome (Fig. 4a, d). Interestingly, the proteome profile after standard cell culture also shows a slight difference to the reference profile, although this shift is much smaller than compared to the single-cell colony profile. The complete segregation of the single-cell colony profile as well as the close proximity of reference and standard cell culture profile can also be seen for AsPC-1, when considering component 1 and 2, thereby representing 22% and 17% of explained variance (Fig. 4b). For component 1 and 3 (only representing 16% and 16% of explained variance), the standard cell culture profile is not completely segregated from the single-cell colony profile but still shows the same tendency (Fig. 4e).

For PANC-1, the complete segregation of the single-cell colony profile from the reference and the standard cell culture profile is also evident, when considering component 1 and 3, which represents a higher explained variance (29% and 21%) than if considering component 1 and 2 (21% and 8%) (Fig. 4c, f). For this cell line, the reference profile shows a complete overlap with the standard cell culture profile.

In addition, unsupervised principal component analysis (PCA) corroborates the previously described findings (Sup. Fig. 6). In almost all PCA plots, the reference and the standard cell culture samples cluster together, while the single-cell colony (SCC) samples clearly segregate from the reference/standard cluster if not even forming a completely separated cluster (Sup. Fig. 6a, b, d, e, f). Only when considering components 1 and 2 for PANC-1 (Sup. Fig. 6c), the different conditions do not show a clear separation as it was already observed in the PLSDA.

These observations suggest that clonal isolation has an impact on the global cellular proteome, but that the extent of this impact might be cell line specific.

### Co-abundance clustering

**Fig. 5:**
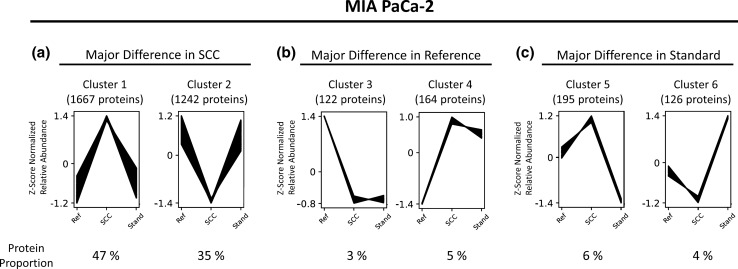
Co-Abundance Cluster Analysis of Identified Proteins from MIA PaCa-2. Depending on the measured intensities in different conditions [Reference (Ref), Single-cell colony (SCC), Standard cell culture (Stand)], the identified proteins were assigned to different co-abundance clusters with a confidence interval of 95%. Cluster assignment was performed using the Clust algorithm. Each line represents an individual protein, while the y-axis illustrates the relative abundance change after *Z*-score normalization. Co-Abundance clusters were sorted into three groups (**a**–**c**), depending on the condition showing the major difference. The number of assigned proteins per cluster is shown above each graph and the corresponding proportion of the total number of assigned proteins is shown below each graph

To evaluate the abundance behavior of the identified proteins across the considered conditions, we performed a co-abundance cluster analysis for each cell line using the publicly available Clust algorithm [35]. The clustering revealed six distinct abundance-courses, with a total of 3516, 5588 and 2673 assigned proteins for MIA PaCa-2 (Fig. 5), AsPC-1 and PANC-1 (Sup. Figs. 7, 8), respectively. The six clusters could be further grouped into three groups, depending on the condition showing the major abundance-change.

For MIA PaCa-2, it is noticeable that the majority of assigned proteins (82%) were assigned to the clusters 1 and 2 and thereby show the major abundance-change in the single-cell colony samples, while the abundance in the reference and the standard cell culture samples behave similar (Fig. 5a). Proteins showing a unique abundance change in the reference (Fig. 5b) or the standard cell culture samples (Fig. 5c) only account for 8% and 10% respectively.

This suggests that clonal isolation could be a trigger for protein-level abundance changes, which however, would have not been triggered by standard cell culture.

Comparable numbers were observed for the cell line PANC-1 with 76% of all proteins showing the major abundance change in the single-cell colony samples (Sup. Fig. 8). However, for AsPC-1, the protein distribution among the clusters is more balanced with 41% of all proteins showing a unique abundance change in the single-cell colony samples, 35% in the reference samples, and 24% in the standard cell culture samples (Sup. Fig. 7). The reason for this may be the fact that for AsPC-1 in total more identified proteins could be assigned to the clusters than for the other two cell lines. Thus, also for AsPC-1, clonal isolation seems to be a potential cause for protein abundance changes affecting a large proportion of the identified proteins.

### Differential expression analysis

To evaluate the proteomic changes upon clonal isolation on single-protein level, we performed a multigroup limma approach for pairwise statistical testing to identify differentially expressed proteins after clonal isolation and after standard cell culture.

**Fig. 6:**
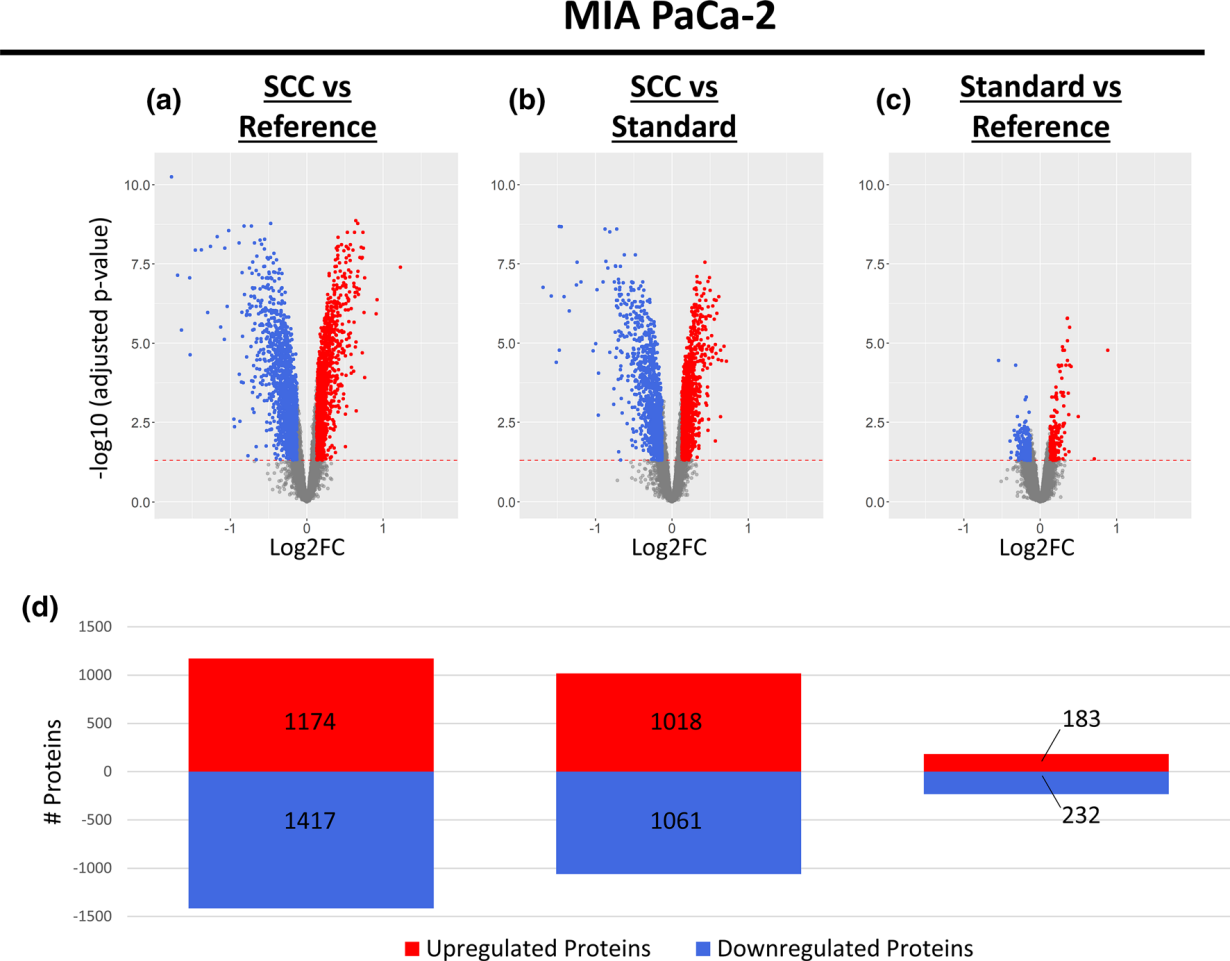
Differential Expression Analysis for MIA PaCa-2 undergoing Clonal Isolation or Standard Cell Culture. For differential expression analysis, a pairwise multigroup limma approach was used to compare the conditions **a** “Single-Cell Culture (SCC) vs Reference”, **b** “Single-Cell Culture (SCC) vs Standard Cell Culture” and **c** “Standard Cell Culture vs Reference” while results were illustrated as volcano plots. The log2 fold changes (log2FC) are plotted on the *x*-axis and corresponding adjusted *p* values in − log10 scale are shown on the *y*-axis. The applied adjusted *p* value cut-off was set to 0.05 (1.3 in − log10 scale, depicted as dashed horizonal line), while the log2FC cut-off was set to ± 0.13 corresponding to 10% FC. Each plot highlights significantly up- (red) or down-regulated (blue) proteins. Hereby, a log2FC > 0 corresponds to an upregulation in the first-mentioned condition. **d** Numbers of significantly up- and down-regulated proteins for each comparison are illustrated as bar chart

For MIA PaCa-2, a total of 5085 significantly differentially expressed proteins were identified in pairwise comparisons of all conditions (Fig. 6, Sup. File 1). Compared to the reference sample, 2591 significant protein abundance changes were associated with clonal isolation (Fig. 6a), while only 415 were identified after standard cell culture (Fig. 6c). When comparing the single-cell colonies against the standard cell culture (Fig. 6b), and thereby also accounting and compensating for the increased number of cell divisions the single-cell has undergone since the clonal isolation, a similar number of differentially expressed proteins (2079 proteins) was identified than if compared to the reference sample. This suggests that the main trigger for differential protein expression is clonal isolation and not the standard cell culturing. On the contrary, the low number of differentially expressed proteins after standard cell culture corroborates the already observed high similarity to the reference cells in the previous PLSDA analysis (Fig. 4) and suggests an only minor influence of the standard cell culture technique on the cellular proteome.

Although revealing less significantly differentially expressed proteins for AsPC-1 (reduced by factor ~ 2) and PANC-1 (reduced by factor ~ 4) compared to MIA PaCa-2, the same tendencies could be observed, namely providing the highest number of differentially expressed proteins when comparing single-cell colonies to either the reference or to the standard cell culture (Sup. Figs. 9, 10, Sup. File 2 and 3). For all three considered cell lines, this indicates that clonal isolation bears a higher risk of proteome alterations than standard cell culture, which could be further visualised by a heatmap (Sup. Fig. 11). Moreover, in all cell lines, the single-cell colony condition revealed the highest mean protein standard deviation across the replicates, possibly suggesting a rather arbitrary introduction of proteome changes instead of a directed or consistent manner.

While the numbers of up- and down-regulated proteins are almost balanced, the observed dimension of protein abundance changes does not exceed a log2 fold change of 2 (equivalent to a fourfold abundance change). To prevent that we mostly discuss quantitatively minimal changes, only proteins revealing a fold change of more than 10% (corresponds to log2FC =  ± 0.13) were considered as being differentially expressed. Of note, usage of a minimal quantitative alteration in addition to significance testing is often used in quantitative proteome studies [52].

For MIA PaCa-2 and PANC-1, some stress-related proteins were significantly upregulated in single-cell colonies including the heat-shock proteins HSP70, HSC70, HSP90 alpha chain, HSJ-2 and GRP75 (Sup. File 1 and 3). This is consistent with previous transcriptomic findings of induced stress-related genes upon single-cell isolation [20, 21]. For AsPC-1, this is not observable, which highlights a different effect of clonal isolation on the cellular stress level for different cell lines (Sup. File 2).

To validate the observed significant abundance changes from differential expression analysis of the so far described DDA data via multigroup limma, an exemplary pattern of six proteins was additionally monitored in MIA PaCa-2 via targeted parallel reaction monitoring (PRM) and resulting log2 fold changes of both approaches were compared (Sup. Fig. 12). For both considered approaches (DDA/limma and PRM), the log2 fold changes of all considered proteins revealed the same tendency even if the absolute fold changes slightly vary between the different approaches with smaller confidence intervals for the DDA/limma approach. This validates the above mentioned findings and emphasizes the reliability of the differential expression analysis.

### Gene ontology enrichment analysis of differentially expressed proteins

To evaluate if certain biological processes are especially represented within the differentially expressed proteins, we performed a gene ontology (GO) enrichment analysis for the respective up- and down-regulated proteins separately.

**Fig. 7:**
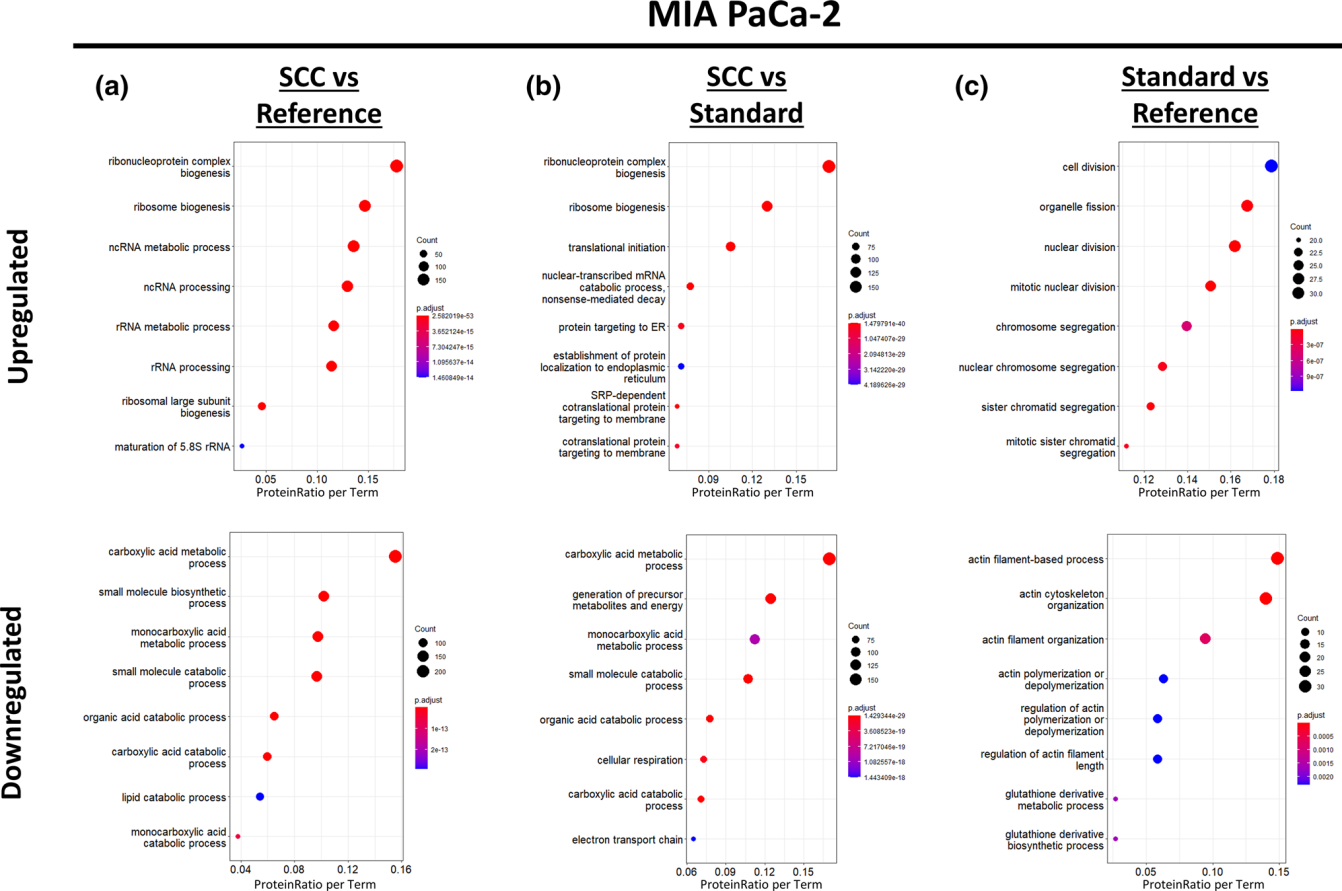
Gene Ontology Enrichment Analysis (Biological Process) of differentially expressed proteins in MIA PaCa-2. Gene Ontology (GO) enrichment analysis of differentially expressed proteins was retrieved using ClusterProfiler in R resulting in dot plots for the comparisons, **a** “Single-Cell Culture (SCC) vs Reference”, **b** “Single-Cell Culture (SCC) vs Standard Cell Culture” and **c** “Standard Cell Culture vs Reference”. The *y*-axis represents the GO terms, while the *x*-axis illustrates the proportion of differentially up- or downregulated proteins that are annotated with the respectively shown GO term (ProteinRatio per Term). The colour of the dots corresponds to the adjusted *p* value of the GO-enrichment and the size of the dots is proportional to the absolute number of differentially expressed proteins enriched in the respective GO-term. The 8 most significantly enriched (adjusted *p* value < 0.05) GO terms in the biological process branch are separately illustrated for up- and downregulated proteins for each comparison

The eight most-enriched GO terms for MIA PaCa-2, AsPC-1 and PANC-1 are shown in Fig. 7, Sup. Figs 13, 14, respectively. We noted that some GO terms are affected in multiple cell lines upon clonal isolation including metabolic processes and cell adhesion, while others appear to be cell line-specific like ribosome biogenesis, translation initiation and DNA replication. Single-cell colonies (SCC) of MIA PaCa-2 and PANC-1 cells show a significant downregulation of metabolic processes (Fig. 7a, b; Sup. Fig. 14a), while AsPC-1 shows the inverse effect revealing a significant upregulation of metabolic processes (Sup. Fig. 13a). These findings could be corroborated by an MTT-based functional cell culture assay monitoring the cellular metabolic activity (Sup. Fig. 15). Such similar behaviour and characteristics of MIA PaCa-2 and PANC-1, while distinguishing from AsPC-1, has already been described in various studies with regard to different cellular aspects [53–55]. This probably reflects their different developmental origin as MIA PaCa-2 and PANC-1 derive from mesenchymal cells whereas AsPC-1 derives from epithelial cells [53].

Single-cell colonies of AsPC-1 and PANC-1 further show a significantly downregulated cell adhesion in our proteomic data (Sup. Figs. 13a, 14b). This was also confirmed by a functional cell culture adhesion assay, where SCC cells show a consistently reduced adhesion for the evaluated extracellular matrix (ECM) substrates compared to the reference (Sup. Fig. 16). Furthermore, also the previously detected increased cell circularity for AsPC-1 (Sup. Fig. 2e) and reduced cell area for PANC-1 (Sup. Fig. 3d) further corroborate our findings as both morphometric parameters suggest a reduced surface attachment and less cell stretching of the respective cells.

Even if the enrichment is not that significant as for single-cell colonies, also the multiply subcultured standard cell culture showed cell line-specific GO terms like cell division, translation, cellular respiration and actin-related processes. As such, standard cell cultured MIA PaCa-2 shows a significant upregulation of cell division and proliferation-associated GO terms compared to the reference (Fig. 7c), which was validated by a cell culture proliferation assay (Sup. Fig. 17a). Furthermore, standard cell cultured PANC-1 cells revealed a significantly downregulated energy metabolism compared to the reference (Sup. Fig. 14c). Assuming that the functional MTT-based cell culture assay primarily monitors the activity of the energy metabolism [56], the thereby derived results for PANC-1 corroborates this proteomic finding (Sup. Fig. 15b).

Generally, it has to be noted that the enrichment of the respective GO terms is less significant for PANC-1 and AsPC-1 as for MIA PaCa-2, which is probably due to the lower number of significantly differentially expressed proteins for PANC-1 and AsPC-1.

The above mentioned findings were further supported by an additional enrichment analysis using STRING, which considers further public databases like KEGG and Reactome in addition to the Gene Ontology Resource. For most of the up- and downregulated protein subsets, the respective network was found to be significantly enriched for protein interactions (PPI enrichment value < 1.0e−16) and analysis against multiple databases (e.g., GO, KEGG, Reactome, UniProtKB Keywords) confirms the previous findings of molecular and functional relevance (Sup. File 4–6).

From the number of differentially expressed protein and the associated biological processes, it is evident that clonal isolation triggers proteome alterations in the form of protein abundance changes. These alterations, however, are associated with different biological processes, depending on the considered cell line, thereby illustrating cell line specificity of the clonal isolation impact.

Furthermore, it should be considered that upon the process of clonal isolation, the cells were expanded to confluent wells and flasks again, before harvesting them. Hence, if the observed proteome alterations were acquired as a single and isolated cell, then these proteome alterations must have been manifested during the subsequent expansion until they were harvested and analysed. Following this, clonal isolation may lead to persistent epigenetic changes, which in turn could lead to the observed proteomic alterations by modifying the respective gene expression. However, further investigations have to be done to validate this hypothesis.

### Repeated limiting dilution cloning further diverges protein profile from reference profile

According to published reports, several rounds of limiting dilution are recommended to ensure monoclonality [57, 58]. In a separate experiment using MIA PaCa-2, we exemplarily tested if repeated clonal isolation using limiting dilution has an either reinforcing or compensating effect on the proteome changes. Therefore, reference cells were compared to cells either undergoing a single round or two successive rounds of limiting dilution. No morphological differences could be observed for doubly-isolated cells on the basis of phase contrast microscopy images (Sup. Fig. 18a), whereas morphometric analysis of fluorescence images revealed a significantly increased nucleus area for doubly-isolate cells at almost constant cell size (Sup. Fig. 18b–d). The acquired proteomic dataset provided a coverage of 11,003–11,537 peptide and 3787–4013 protein identifications per fraction (Sup. Fig. 19a) resulting in a total of 6747 unique proteins and a peptide-level TMT-labelling efficiency of 99.9%.

**Fig. 8:**
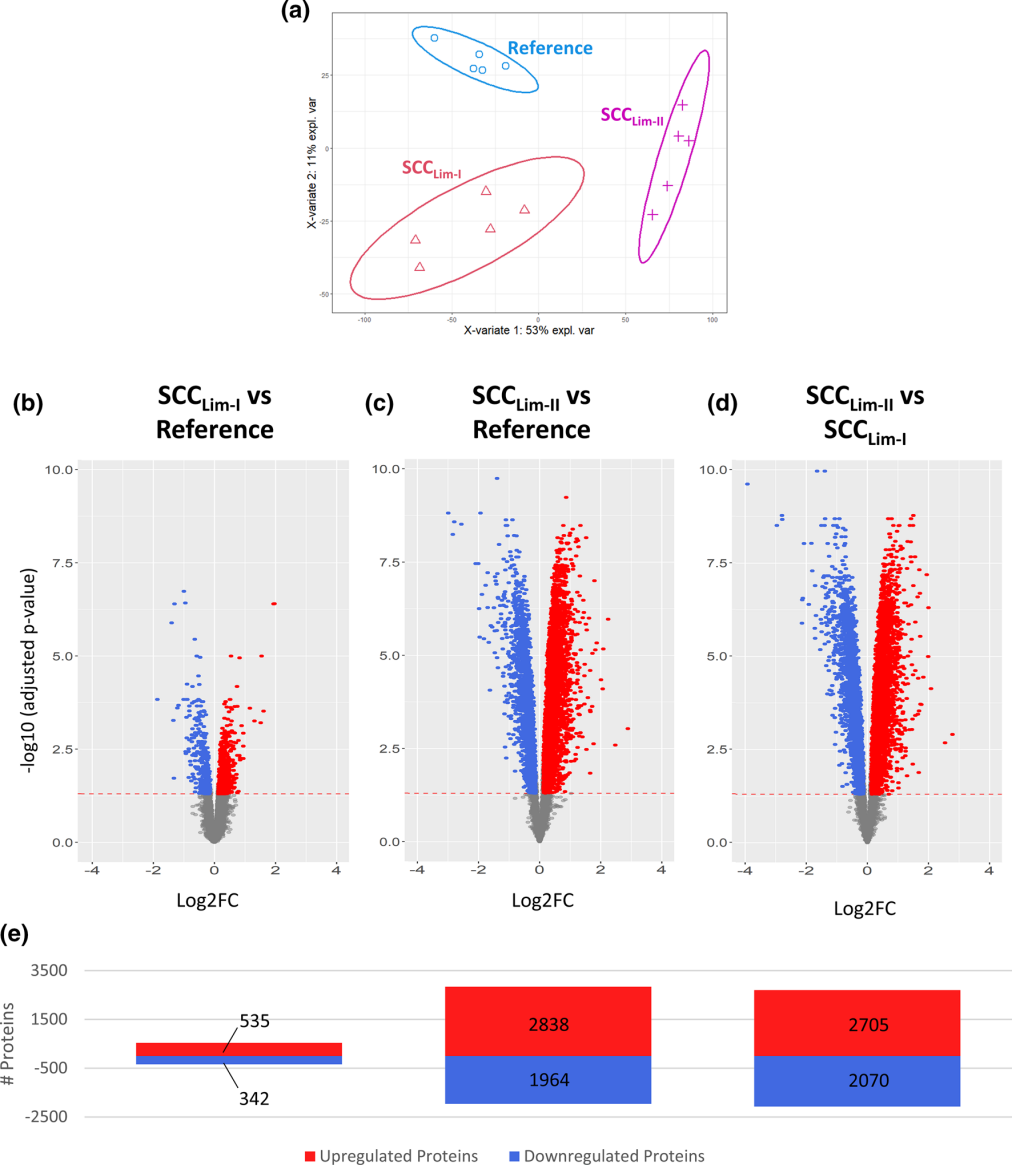
Impact of repeated limiting dilution cloning on the cellular proteome of MIA PaCa-2 cells. MIA PaCa-2 cells were either subjected to a single round (Lim-I) or two successive rounds (Lim-II) of limiting dilution cloning before analysing and comparing the cellular proteomes. **a** Partial Least Squares Discriminant Analysis (PLSDA) of the obtained dataset. Protein profiles of each sample together with the corresponding condition annotation (Reference, SCC_Lim-I_, SCC_Lim-II_) were submitted to the analysis, where *x*- and *y*-axis represent the percentage of explained variance of the respective component. Ellipses represent 95% confidence intervals. Differential Expression Analysis using a pairwise multigroup limma approach to compare the conditions, **b** “SCC_Lim-I_ vs Reference”, **c** “SCC_Lim-II_ vs Reference” and **d** “SCC_Lim-II_ vs SCC_Lim-I_” while results were illustrated as volcano plots. The log2 fold changes (log2FC) are plotted on the x-axis and corresponding adjusted *p* values in − log10 scale are shown on the *y*-axis. The applied adjusted p-value cut-off was set to 0.05 (1.3 in − log10 scale, depicted as dashed horizonal line), while the log2FC cut-off was set to ± 0.13 corresponding to 10% FC. Each plot highlights significantly up- (red) or down-regulated (blue) proteins. Hereby, a log2FC > 0 corresponds to an upregulation in the first-mentioned condition. **e** Numbers of significantly up- and down-regulated proteins for each comparison are illustrated as bar chart

PLSDA of the obtained proteomic data shows a clear segregation of doubly-isolated cells (SCC_Lim-II_) from single-isolated cells (SCC_Lim-I_) on component 1 and further illustrates an even higher divergence of the SCC_Lim-II_ protein profile from the reference profile than SCC_Lim-I_ (Fig. 8a). This is further corroborated by the differential expression analysis, which revealed a higher number of significantly differentially expressed proteins in SCC_Lim-II_ (4802 proteins) than in SCC_Lim-I_ (877 proteins) when individually compared to the reference sample (Fig. 8b, c, e; Sup. File 7). Also in the heatmap representation, SCC_Lim-II_ shows a more heterogeneous abundance distribution with the highest mean protein standard deviation across the replicates (Sup. Fig. 20a). When directly comparing SCC_Lim-II_ against SCC_Lim-I_, the comparatively high number of significantly differentially expressed proteins (4775 proteins) suggests that a repeated round of limiting dilution introduces additional proteome changes (Fig. 8d, e).

Furthermore, the aforementioned stress-related heat-shock proteins HSP70, HSC70, HSP90 and HSJ-2 could also be found to be significantly upregulated in SCC_Lim-II_, even when compared to SCC_Lim-I_ (Sup. File 7). This suggests an enhanced induction of stress-related proteins by the second round of limiting dilution.

**Fig. 9:**
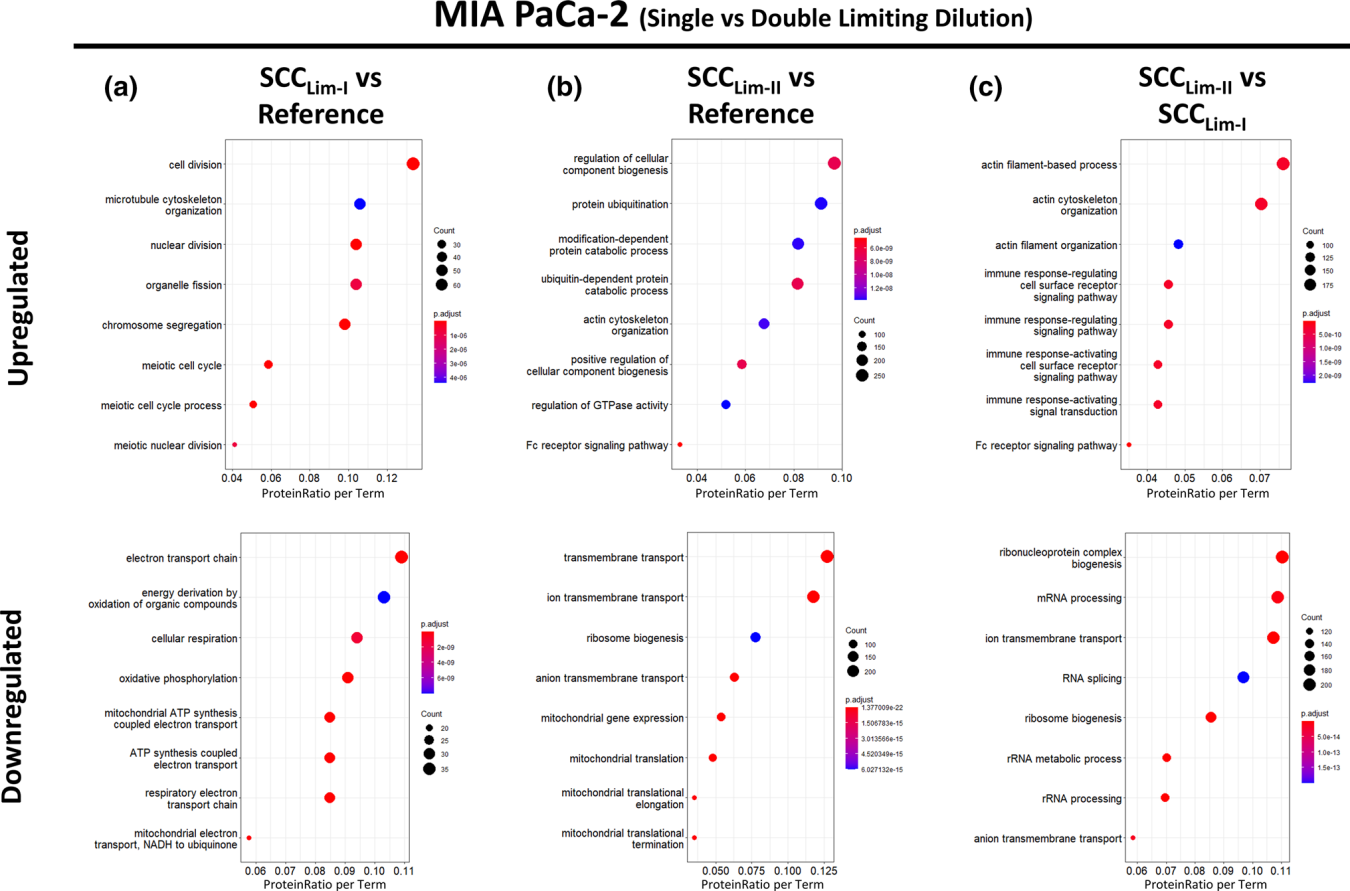
Gene Ontology Enrichment Analysis (Biological Process) of differentially expressed proteins in single- and doubly-isolated MIA PaCa-2 cells. MIA PaCa-2 cells were either subjected to a single round (Lim-I) or two successive rounds (Lim-II) of limiting dilution cloning. Gene Ontology (GO) enrichment analysis of differentially expressed proteins was retrieved using ClusterProfiler in R resulting in dot plots for the comparisons, **a** “SCC_Lim-I_ vs Reference”, **b** “SCC_Lim-II_ vs Reference” and **c** “SCC_Lim-II_ vs SCC_Lim-I_”. The *y*-axis represents the GO terms, while the *x*-axis illustrates the proportion of differentially up- or downregulated proteins that are annotated with the respectively shown GO term (ProteinRatio per Term). The colour of the dots corresponds to the adjusted *p* value of the GO-enrichment and the size of the dots is proportional to the absolute number of differentially expressed proteins enriched in the respective GO-term. The 8 most significantly enriched (adjusted *p* value < 0.05) GO terms in the biological process branch are separately illustrated for up- and downregulated proteins for each comparison

Considering the associated GO terms, different terms for SCC_Lim-II_ and SCC_Lim-I_ were enriched, both for the individual comparison with the reference (Fig. 9a, b) as well as for the comparison against each other (Fig. 9c). This suggests that the introduced proteome changes during the second round of limiting dilution are rather additional changes instead of enhancing the already existing changes. The renewed observation of a significantly downregulated metabolism when comparing SCC_Lim-I_ against the reference, independently validates the results from the previous experiment (Fig. 7). Additionally, the dataset provides an upregulation of proliferation-associated terms like cell division and nuclear division when compared to the reference, which has not emerged among the top eight enriched GO terms in the previous experiment. Both observations could be validated by functional cell culture assays monitoring proliferation and metabolic activity (Sup. Fig. 17), which highlights the robustness of our findings.

In general, the described findings support the hypothesis that limiting dilution is the cause of the observed proteome changes and that each round further modifies the cellular proteome. Furthermore, the fact that an additional round of clonal isolation further reinforces the proteome changes points towards persistence of previously introduced alterations. However, further investigations are required to validate the long-term persistence of the observed proteome changes.

### *Single-cell isolation *via* FACS introduces different proteome changes*

An alternative method to limiting dilution for single-cell isolation is fluorescence-activated cell sorting (FACS). To evaluate if the proteome changes introduced by FACS-assisted single-cell sorting are comparable or different to cells subjected to limiting dilution cloning, an additional experiment was exemplarily performed with MIA PaCa-2 cells. Therefore, reference cells were compared to cells undergoing a single round of clonal isolation but with different methods including limiting dilution or FACS. For FACS-isolated cells, no morphological or morphometric differences could be observed on the basis of phase contrast and fluorescence microscopy images (Sup. Fig. 18). The resulting proteomic dataset revealed the identification and quantification of 11,313–12,361 peptides and 3791–4166 proteins per fraction (Sup. Fig. 19b) leading to a total of 6855 unique proteins with a TMT-labelling efficiency of 99.8%.

**Fig. 10:**
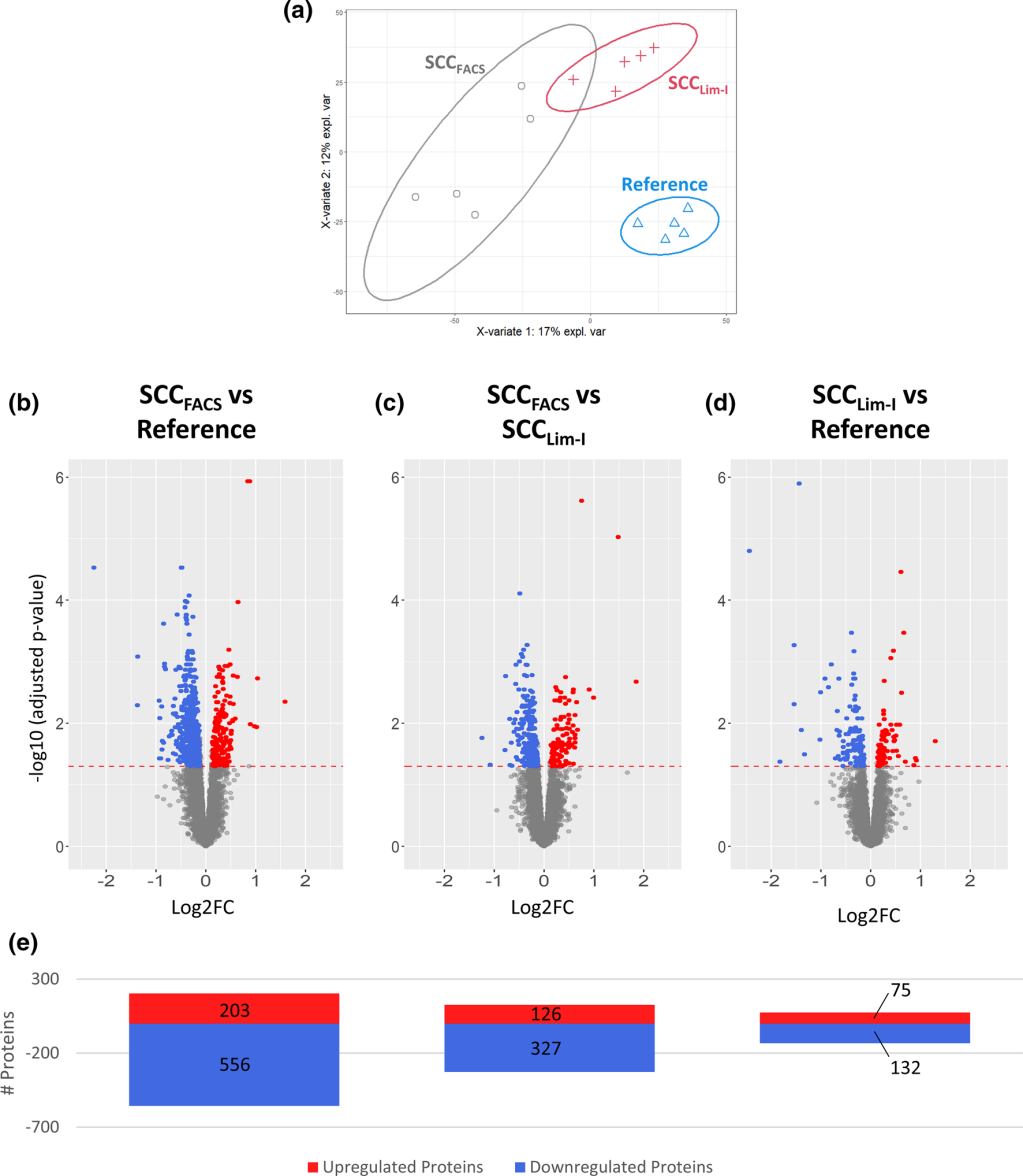
Method-specific impact of Single-Cell Isolation via Limiting Dilution or FACS on the Cellular Proteome of MIA PaCa-2 cells. MIA PaCa-2 cells were subjected to a single round of clonal isolation either using limiting dilution or FACS-assisted single-cell isolation before analysing and comparing the cellular proteomes. **a** Partial Least Squares Discriminant Analysis (PLSDA) of the obtained dataset. Protein profiles of each sample together with the corresponding condition annotation (Reference, SCC_Lim-I_, SCC_FACS_) were submitted to the analysis, where *x*- and *y*-axis represent the percentage of explained variance of the respective component. Ellipses represent 95% confidence intervals. Differential Expression Analysis using a pairwise multigroup limma approach to compare the conditions **b** “SCC_FACS_ vs Reference”, **c** “SCC_FACS_ vs SCC_Lim-I_” and **d** “SCC_Lim-I_ vs Reference” while results were illustrated as volcano plots. The log2 fold changes (log2FC) are plotted on the *x*-axis and corresponding adjusted *p* values in − log10 scale are shown on the *y*-axis. The applied adjusted *p* value cut-off was set to 0.05 (1.3 in − log10 scale, depicted as dashed horizonal line), while the log2FC cut-off was set to ± 0.13 corresponding to 10% FC. Each plot highlights significantly up- (red) or down-regulated (blue) proteins. Hereby, a log2FC > 0 corresponds to an upregulation in the first-mentioned condition. **e** Numbers of significantly up- and down-regulated proteins for each comparison are illustrated as bar chart

A global exploration of the dataset with PLSDA revealed a clear segregation of FACS-isolated cells (SCC_FACS_) and cells isolated via limiting dilution (SCC_Lim-I_) from the reference protein profile, with even comparable distance (Fig. 10a). Also the heatmap representation of the dataset shows a comparable increase in abundance heterogeneity and mean protein standard deviation for SCC_Lim-I_ and SCC_FACS_ compared to the reference (Sup. Fig. 20b). However, even if the 95% confidence interval of the PLSDA shows some overlap between SCC_FACS_ and SCC_Lim-I_, the clearly different orientations of the respective clusters illustrate method-specific effects on the proteome (Fig. 10a). This is supported by the differential expression analysis, which provides 759 differentially expressed proteins for SCC_FACS_ compared to the reference, but also 453 proteins when comparing SCC_FACS_ against SCC_Lim-I_ (Fig. 10b, c; Sup. File 8).

Stress-related heat-shock proteins HSP70 and HSJ-1 could also be found to be significantly upregulated in SCC_FACS_ when compared to the reference, while only HSP70 appears to be significantly upregulated when compared to SCC_Lim-I_ (Sup. File 8). This suggests FACS to induce slightly less stress-related proteins than limiting dilution, which is in contrast to the literature suggesting limiting dilution to cause less cellular stress [11].

**Fig. 11:**
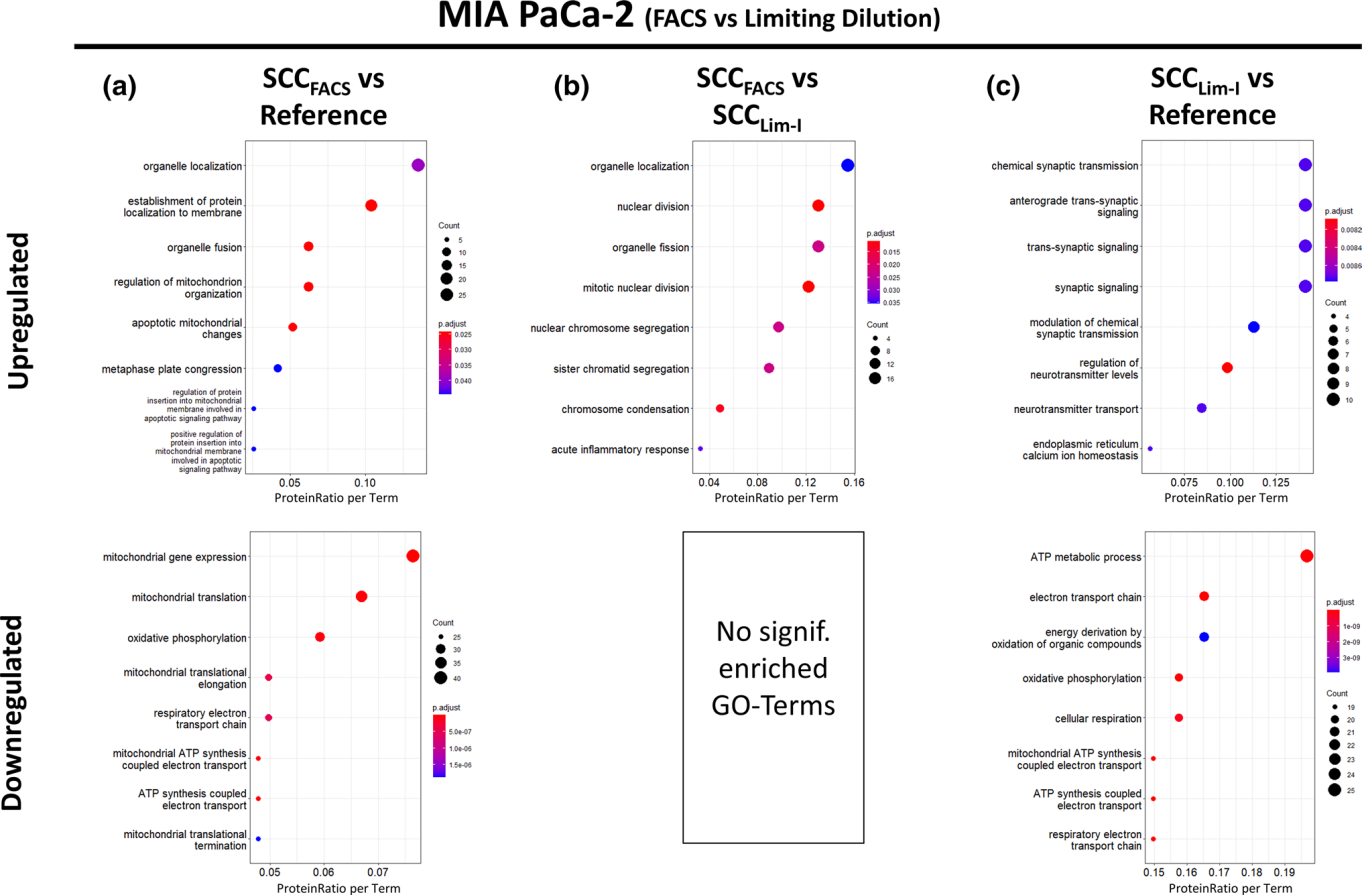
Gene Ontology Enrichment Analysis (Biological Process) of differentially expressed proteins in MIA PaCa-2 cells undergoing Single-Cell Isolation via Limiting Dilution or FACS. MIA PaCa-2 cells were subjected to a single round of clonal isolation either using limiting dilution or FACS-assisted single-cell isolation. Gene Ontology (GO) enrichment analysis of differentially expressed proteins was retrieved using ClusterProfiler in R resulting in dot plots for the comparisons **a** “SCC_FACS_ vs Reference”, **b** “SCC_FACS_ vs SCC_Lim-I_” and **c** “SCC_Lim-I_ vs Reference”. The *y*-axis represents the GO terms, while the *x*-axis illustrates the proportion of differentially up- or downregulated proteins that are annotated with the respectively shown GO term (ProteinRatio per Term). The colour of the dots corresponds to the adjusted p-value of the GO-enrichment and the size of the dots is proportional to the absolute number of differentially expressed proteins enriched in the respective GO-term. The 8 most significantly enriched (adjusted *p* value < 0.05) GO terms in the biological process branch are separately illustrated for up- and downregulated proteins for each comparison

The method-specific effects also become apparent when considering the associated enriched GO terms, which show different terms for each method compared to the reference (Fig. 11a, c) as well as when compared against each other (Fig. 11b). Interestingly, the comparison SCC_FACS_ vs Reference as well as SCC_Lim-I_ vs Reference both show a significant downregulation of metabolic activity, especially energy metabolism, which again corroborates previous findings from an independent experiment and, apart from the method-specific effects, also suggests a joint and similar effect of both methods on the cellular proteome (Fig. 11a, c). This was also validated by the MTT-based functional cell culture assay, showing a reduced metabolic activity for SCC_FACS_ and SCC_Lim-I_ compared to the reference (Sup. Fig. 17b). Considering metabolic activity as a measure for cell viability, this finding is in accordance to literature, where FACS-assisted single-cell isolation was reported to negatively affect cell viability [10]. Interestingly, both methods for single-cell isolation show a comparable extent of reducing cell viability with no significant difference when comparing SCC_Lim-I_ against SCC_FACS_ (Sup. Fig. 17b). Furthermore, also the observed upregulation of proliferation-associated GO terms in SCC_FACS_ compared to SCC_Lim-I_ could be seen and validated by the respective functional cell culture assay (Sup. Fig. 17a).

Thus, this data show that each round of clonal isolation introduces proteome changes irrespective of the used method for single-cell isolation. We both observed method-specific changes as well as a reduced metabolic activity as a common pathway, affected by both methods. As this common effect was reproducible and independent of the used method, this possibly reflects a predisposition of the used cell line, here MIA PaCa-2, which tends to reduce energy metabolism and increase proliferation when exposed to the stress of single-cell isolation. However, as MIA PaCa-2 is a pancreatic cancer cell line and assuming that the functional MTT-based cell culture assay primarily monitors the mitochondrial energy metabolism [56], it is also conceivable that the increased proliferation of MIA PaCa-2 single-cell colonies requires the increased usage of the Warburg effect [59, 60] and thereby shows reduced respiratory energy metabolism. However, this requires further experimental validation.

Across all experiments, our data repeatedly revealed cell-line specific effects of clonal isolation on the respective cellular proteome. Although the global introduction of proteome changes upon clonal isolation appears to be rather arbitrary, each investigated cell line apparently revealed different biological processes which are particularly vulnerable to proteome changes. Although no generally applicable statement with validity for all cell lines can be derived, it becomes apparent that the impact of clonal isolation requires individual investigation or at least consideration when interpreting data stemming from experiments that include this step.

It is important to consider that clonal isolation can add a systematic bias to gene editing experiments by additionally adding unintended proteome changes. However, the here observed cell line specific effects on proteome level open the possibility to minimize this bias by comparing different cell lines and potentially find a cell line showing the least possible vulnerability for proteome changes upon clonal isolation. This, however, requires further investigations and the systematic comparison of a variety of cell lines of different entity.

## Conclusion

This study shows that clonal isolation can have an impact on the cellular proteome. However, the extent and the biological processes that are affected seem to be cell line- and method-specific. In this study, we could show a different impact of clonal isolation on mesenchymal-derived cell lines and epithelial-derived cell lines. While the clonal isolation of the mesenchymal-derived cell lines MIA PaCa-2 and PANC-1 revealed a downregulated metabolism in the presence of upregulated protein representatives for cell stress, the epithelial-derived cell line AsPC-1 showed an upregulated metabolism without significant abundance changes of the respective cell stress proteins. Furthermore, we observed a reproducible and method-independent reduction of metabolic activity together with an increased proliferation after clonal isolation for MIA PaCa-2 and thereby possibly represent the cell line specific vulnerability of this cell line.

We assume that this is of interest for the whole field of genomic editing, where clonal isolation is a mandatory part of the overall workflow. Consequently, it is important to consider that clonal isolation can add a systematic bias to gene editing experiments by additionally introducing unintended proteome changes. The observed cell line specific effects open the possibility for future experiments to potentially find a cell line being as little vulnerable to proteome changes upon clonal isolation as possible and thereby to minimize this bias. However, as this study investigated three adherent pancreatic cancer cell lines, further investigations with additional cell lines of different entity are needed to gain further insights and to potentially control the cellular impact of clonal isolation.

## Conflict of interest

The authors have no conflict of interest to declare that are relevant to the content of this article.

## Supplementary Information

Below is the link to the electronic supplementary material.

Supplementary file1 (XLSX 2232 KB)

Supplementary file2 (XLSX 1991 KB)

Supplementary file3 (XLSX 1857 KB)

Supplementary file4 (XLSX 2655 KB)

Supplementary file5 (XLSX 762 KB)

Supplementary file6 (XLSX 347 KB)

Supplementary file7 (XLSX 2621 KB)

Supplementary file8 (XLSX 2065 KB)

**Sup. Fig. 1 Single-Cell Isolation of Human Pancreatic Cell Lines via Limiting Dilution.** Human pancreatic cells including a) MIA PaCa-2, b) AsPC-1 and c) PANC-1 were cultured in 96-well flat-bottom plates. Upon limiting dilution, 96-well plates were regularly inspected via light microscopy for emerging single-cell colonies, which could be first observed after 24 hours for MIA PaCa-2 and AsPC-1 and after 72 hours for PANC-1. These single-cell colonies resulted in a spherical colony after 20 days for MIA PaCa-2 and AsPC-1 and after 10 days for PANC-1. The particular size of the scale bar is indicated in each image and has been previously calibrated for each magnification using a Neubauer counting chamber. Contrast and brightness have been adjusted (TIF 15463 KB)

**Sup. Fig. 2 Cell Morphology before and after Standard Cell Culture or Clonal Isolation of human pancreatic AsPc-1 cell line.** Human pancreatic AsPc-1 reference cells either undergoing standard cell culture or clonal isolation via limiting dilution to obtain single-cell colonies. (a) Cells were cultured in cell culture flasks and imaged via phase contrast microscopy (5x and 20x magnification). (b) Cells were fluorescence stained for F-actin by fluorophore labelled Phalloidin (green) and dsDNA by Hoechst 33342 (blue). Maximum intensity projection of 40x z-stack images is shown. (c–e) Morphometric parameters of individual cells were analysed based on Phalloidin and Hoechst 33342 fluorescence staining. Three independent replicates and at least 388 nuclei (c) or 100 cells (d,e) per condition and replicate were analysed. Scatter plot dots represent mean values per replicate (error bars show mean and S.E.M., * – p<0.05, ns – non significant) (TIF 7076 KB)

**Sup. Fig. 3 Cell Morphology before and after Standard Cell Culture or Clonal Isolation of human pancreatic PANC-1 cell line.** Human pancreatic PANC-1 reference cells either undergoing standard cell culture or clonal isolation via limiting dilution to obtain single-cell colonies. (a) Cells were cultured in cell culture flasks and imaged via phase contrast microscopy (5x and 20x magnification). (b) Cells were fluorescence stained for F-actin by fluorophore labelled Phalloidin (green) and dsDNA by Hoechst 33342 (blue). Maximum intensity projection of 40x z-stack images is shown. (c–e) Morphometric parameters of individual cells were analysed based on Phalloidin and Hoechst 33342 fluorescence staining. Three independent replicates and at least 272 nuclei (c) or 100 cells (d,e) per condition and replicate were analysed. Scatter plot dots represent mean values per replicate (error bars show mean and S.E.M., ** – p<0.01, *** – p<0.001, ns – non significant) (TIF 7285 KB)

**Sup. Fig. 4 Proteomic Data Characteristics including Number of Identified Peptides and Proteins and Labelling Efficiency.** For peptide and protein identification, acquired LC-MS/MS data was searched against a human database using MaxQuant with 1 % false discovery rate (FDR). (a) Resulting numbers of identified peptides and proteins are shown for each fraction (F1–F12) and cell line (MIA PaCa-2, AsPC-1, PANC-1). (b) The efficiency of peptide-level TMT-labelling was evaluated by calculating the intensity-ratio of N-terminally labelled, tryptic peptides against all identified tryptic peptides (TIF 889 KB)

**Sup. Fig. 5 Applicability Validation of TMTpro-16plex using a HEK-E.Coli Benchmark Dataset.** (a) The TMTpro-16plex benchmark dataset was created by adding different amounts of *E.coli* peptides (0.5 µg, 1.5 µg, 4 µg) to a consistent amount of human HEK peptides (20 µg), resulting in one “Hek only” condition and three mixtures of know peptide mass ratios (*E.coli*:HEK 1:50, 1:17, 1:6) with four replicates each. After isobaric labelling with tandem mass tag (TMTpro-16plex), all samples were pooled and offline fractionated using high-pH reversed phase chromatography. Resulting fractions were subjected to mass spectrometric analysis operated in data-dependent acquisition mode (DDA). Database search of resulting raw data was performed using MaxQuant with subsequent assignment to *E.Coli* and HEK proteins. (b) Protein intensities after median normalization with MSstatsTMT are illustrated in log2 scale for each TMT channel of the mixtures in the *E.coli* and the HEK fraction as boxplots, representing corresponding protein abundance. Lower and upper box boundaries represent the 25^th^ and 75^th^ percentiles, line inside the box represent median, upper and lower error lines represent 1.5 times the interquartile range and circles represent data points exceeding that error range. (c) Differential expression analysis between mixtures was performed using a pairwise multigroup limma approach. The distribution of resulting fold changes for each comparison (“Mix-2 vs Mix-1”, “Mix-3 vs Mix-2”, “Mix-3 vs Mix-2”) are illustrated in log2 scale as density plots. For the *E.coli* fraction, the observed median log2 fold change and the therefrom derived delogarithmized median fold change ratio are stated below each comparison plot. Together with the expected fold change based on the known mass ratio of added *E.coli* peptides (3:1, 2.7:1, 8:1), the respective ratio compression factor could be calculated (TIF 2610 KB)

**Sup. Fig. 6 Principal Component Analysis (PCA).** Protein profiles of each sample were submitted to PCA analysis. For the analysis either components 1 and 2 (a,b,c) or components 1 and 3 (d,e,f) were considered, where x- and y-axis represent the percentage of explained variance of the respective component (TIF 3560 KB)

**Sup. Fig. 7 Co-Abundance Cluster Analysis of Identified Proteins from AsPC-1.** Depending on the measured intensities in different conditions (Reference (Ref), Single-cell colony (SCC), Standard cell culture (Stand)), the identified proteins were assigned to different co-abundance cluster with a confidence interval of 95 %. Cluster assignment was performed using the Clust algorithm. Each line represents an individual protein, while the y-axis illustrates the relative abundance change after Z-score normalization. Co-Abundance clusters were sorted into three groups (a,b,c), depending on the condition showing the major difference. The number of assigned proteins per cluster is shown above each graph and the corresponding proportion of the total number of assigned proteins is shown below each graph (TIF 1498 KB)

**Sup. Fig. 8 Co-Abundance Cluster Analysis of Identified Proteins from PANC-1.** Depending on the measured intensities in different conditions (Reference (Ref), Single-cell colony (SCC), Standard cell culture (Stand)), the identified proteins were assigned to different co-abundance cluster with a confidence interval of 95 %. Cluster assignment was performed using the Clust algorithm. Each line represents an individual protein, while the y-axis illustrates the relative abundance change after Z-score normalization. Co-Abundance clusters were sorted into three groups (a,b,c), depending on the condition showing the major difference. The number of assigned proteins per cluster is shown above each graph and the corresponding proportion of the total number of assigned proteins is shown below each graph (TIF 1455 KB)

**Sup. Fig. 9 Differential Expression Analysis for AsPC-1 undergoing Clonal Isolation or Standard Cell Culture.** For differential expression analysis, a pairwise multigroup limma approach was used to compare the conditions a) “Single-Cell Culture (SCC) vs Reference”, b) “Single-Cell Culture (SCC) vs Standard Cell Culture” and c) “Standard Cell Culture vs Reference” while results were illustrated as volcano plots. The log2 fold changes (log2FC) are plotted on the x-axis and corresponding adjusted p-values in -log10 scale are shown on the y-axis. The applied adjusted p-value cut-off was set to 0.05 (1.3 in -log10 scale, depicted as dashed horizonal line), while the log2FC cut-off was set to +/- 0.13 corresponding to 10 % FC. Each plot highlights significantly up- (red) or down-regulated (blue) proteins. Hereby, a log2FC > 0 corresponds to an upregulation in the first-mentioned condition. (d) Numbers of significantly up- and down-regulated proteins for each comparison are illustrated as bar chart (TIF 1215 KB)

**Sup. Fig. 10 Differential Expression Analysis for PANC-1 undergoing Clonal Isolation or Standard Cell Culture.** For differential expression analysis, a pairwise multigroup limma approach was used to compare the conditions a) “Single-Cell Culture (SCC) vs Reference”, b) “Single-Cell Culture (SCC) vs Standard Cell Culture” and c) “Standard Cell Culture vs Reference” while results were illustrated as volcano plots. The log2 fold changes (log2FC) are plotted on the x-axis and corresponding adjusted p-values in -log10 scale are shown on the y-axis. The applied adjusted p-value cut-off was set to 0.05 (1.3 in -log10 scale, depicted as dashed horizonal line), while the log2FC cut-off was set to +/- 0.13 corresponding to 10 % FC. Each plot highlights significantly up- (red) or down-regulated (blue) proteins. Hereby, a log2FC > 0 corresponds to an upregulation in the first-mentioned condition. (d) Numbers of significantly up- and down-regulated proteins for each comparison are illustrated as bar chart (TIF 1152 KB)

**Sup. Fig. 11 Heatmap Representation of Cell Line Specific Protein Expression Profiles and Mean Protein Standard Deviation per Condition.** Acquired proteomic data for MIA PaCa-2, AsPC-1 and PANC-1 were visualised via heatmap representing proteins as rows, conditions (Reference, Standard, Single-cell colony SCC) as columns and the respective protein abundance as colour-coded field. Samples from similar conditions were grouped together and indicated accordingly above the heatmap. The mean protein standard deviation (SD) was calculated for each condition individually by determining the standard deviation of each protein across the 5 respective replicates before calculating the mean over all protein standard deviations. The highest mean protein standard deviation for each cell line is highlighted in bold (TIF 3438 KB)

**Sup. Fig. 12 Comparison of log2 fold changes from data-dependent acquisition (DDA) mode and targeted parallel reaction monitoring (PRM) mode in MIA PaCa-2.** Labelled or unlabelled peptide samples were analysed in DDA- or PRM-mode and subjected to pairwise comparisons using multigroup limma or two-sample t-test, respectively. Resulting log2 fold changes for the DDA/Limma- (orange) and PRM/t-test-approach are shown as bar plots for 6 exemplary proteins (with corresponding Uniprot-IDs) for the comparisons a) “Single-Cell Culture (SCC) vs Reference” and b) “Single-Cell Culture (SCC) vs Standard Cell Culture”. Error bars correspond to the 95 % confidence intervals (TIF 618 KB)

**Sup. Fig. 13 Gene Ontology Enrichment Analysis (Biological Process) of differentially expressed proteins in AsPC-1.** Gene Ontology (GO) enrichment analysis of differentially expressed proteins was retrieved using ClusterProfiler in R resulting in dot plots for the comparisons a) “Single-Cell Culture (SCC) vs Reference”, b) “Single-Cell Culture (SCC) vs Standard Cell Culture” and c) “Standard Cell Culture vs Reference”. The y-axis represents the GO terms, while the x-axis illustrates the proportion of differentially up- or downregulated proteins that are annotated with the respectively shown GO term (ProteinRatio per Term). The colour of the dots corresponds to the adjusted p-value of the GO-enrichment and the size of the dots is proportional to the absolute number of differentially expressed proteins enriched in the respective GO-term. The 8 most significantly enriched (adjusted p-value < 0.05) GO terms in the biological process branch are separately illustrated for up- and downregulated proteins for each comparison (TIF 6488 KB)

**Sup. Fig. 14 Gene Ontology Enrichment Analysis (Biological Process) of differentially expressed proteins in PANC-1.** Gene Ontology (GO) enrichment analysis of differentially expressed proteins was retrieved using ClusterProfiler in R resulting in dot plots for the comparisons a) “Single-Cell Culture (SCC) vs Reference”, b) “Single-Cell Culture (SCC) vs Standard Cell Culture” and c) “Standard Cell Culture vs Reference”. The y-axis represents the GO terms, while the x-axis illustrates the proportion of differentially up- or downregulated proteins that are annotated with the respectively shown GO term (ProteinRatio per Term). The colour of the dots corresponds to the adjusted p-value of the GO-enrichment and the size of the dots is proportional to the absolute number of differentially expressed proteins enriched in the respective GO-term. The 8 most significantly enriched (adjusted p-value < 0.05) GO terms in the biological process branch are separately illustrated for up- and downregulated proteins of each comparison (TIF 4045 KB)

**Sup. Fig. 15 Evaluation of cellular metabolic activity by colorimetric MTT-based cell culture assay.** Human pancreatic reference cells (MIA PaCa-2, PANC-1, AsPC-1) either undergo standard cell culture (Standard) or clonal isolation via limiting dilution (SCC). For each condition, 5,000 cells/well of the respective cell line were seeded and cultured for 48 hours before analysing metabolic activity with colorimetric MTT-based assay at 570 nm (reference wavelength 630 nm). Each condition was performed with n=7 independent replicates. The bar chart shows the mean of blank-corrected absorbance values and corresponding standard deviation as error bars. One-way ANOVA with Tukey´s multiple comparisons test was used. Statistical significance was defined as ** - p<0.01, **** - p<0.0001, ns – non significant (TIF 412 KB)

**Sup. Fig. 16 Evaluation of cell adhesion capability by colorimetric extracellular matrix (ECM) adhesion assay.** Human pancreatic reference cells (PANC-1, AsPC-1) either undergo standard cell culture (Standard) or clonal isolation via limiting dilution (SCC). For each condition, 150,000 cells of the respective cell line were added to each precoated well and incubated for 2 hours before analysing cell adhesion capability by colorimetric ECM adhesion assay at 570 nm. Each condition was performed in duplicates. The bar chart shows the mean of blank-corrected absorbance values and corresponding standard deviation as error bars. One-way ANOVA with Dunnett´s multiple comparisons test was used. Statistical significance to the reference was defined as * - p<0.05, ** - p<0.01, *** - p<0.001, while unlabeled bars correspond to non-significant changes (TIF 557 KB)

**Sup. Fig. 17 Evaluation of proliferation and metabolic activity of MIA PaCa-2 cells.** Human MIA PaCa-2 reference cells either undergo standard cell culture (Standard), clonal isolation via a single round of limiting dilution (SCC_Lim-I_), two successive rounds of limiting dilution (SCC_Lim-II_) or clonal isolation via FACS-assisted single-cell isolation (SCC_FACS_). For each condition, 5,000 cells/well were seeded and cultured for 48 hours before either analysing proliferation with colorimetric BrdU-incorporation ELISA-assay at 370 nm (492 nm reference wavelength) or metabolic activity with colorimetric MTT-based assay at 570 nm (reference wavelength 630 nm). Each condition was performed with n=7 independent replicates. The bar chart shows the mean of blank-corrected absorbance values and corresponding standard deviation as error bars. Metabolic activity data for the conditions “Reference”, “Standard” and “SCC_Lim-I_” is identical to the data shown in Sup. Fig. 15 – a and is repeated for comparability reasons. One-way ANOVA with Tukey´s multiple comparisons test was used. Statistical significance was defined as ** - p<0.01, **** - p<0.0001, ns – non significant (TIF 520 KB)

** Sup. Fig. 18 Cell Morphology before and after Clonal Isolation of human pancreatic MIA PaCa-2 cells via two rounds of limiting dilution or via FACS-assisted single-cell isolation.** Human pancreatic MIA PaCa-2 reference cells either undergoing clonal isolation by two successive rounds of limiting dilution (SCC_Lim-II_) or by one round of FACS-assisted single-cell isolation (SCC_FACS_). (a) Cells were cultured in cell culture flasks and imaged via phase contrast microscopy (5x and 20x magnification). (b) Cells were fluorescence stained for F-actin by fluorophore labelled Phalloidin (green) and dsDNA by Hoechst 33342 (blue). Maximum intensity projection of 40x z-stack images is shown. (c-e) Morphometric parameters of individual cells were analysed based on Phalloidin and Hoechst 33342 fluorescence staining. Three independent replicates and at least 216 nuclei (c) or 100 cells (d,e) per condition and replicate were analysed. Data for MIA PaCa-2 reference are identical to the data shown in Fig. 2 and is repeated for comparability reasons. Experimental and statistical analysis was performed together for all 5 MIA PaCa-2 cell lines. Scatter plot dots represent mean values per replicate (error bars show mean and S.E.M., * - p<0.05, ns – non significant) (TIF 7112 KB)

**Sup. Fig. 19 Proteomic Data Characteristics for datasets investigating repeated limiting dilution cloning and clonal isolation using FACS.** Proteomes of human pancreatic MIA PaCa-2 reference cells were either compared to the proteome of (a) cells undergoing a single round or two successive rounds of limiting dilution or to the proteome of (b) cells undergoing a single round of clonal isolation but with different methods including limiting dilution or FACS. For peptide and protein identification, acquired LC-MS/MS data were searched against a human database using MaxQuant with 1 % false discovery rate (FDR). Resulting numbers of identified peptides and proteins of respective datasets are shown for each fraction (F1–F12) as well as the efficiency of peptide-level TMT-labelling, which was evaluated by calculating the intensity-ratio of N-terminally labelled, tryptic peptides against all identified tryptic peptides (TIF 796 KB)

**Sup. Fig. 20 Heatmap Representation of Method Specific Protein Expression Profiles and Mean Protein Standard Deviation per Condition.** Acquired proteomic datasets for MIA PaCa-2 either (a) undergoing several rounds of limiting dilution or (b) undergoing a single round of clonal isolation with different methods were visualised via heatmap representing proteins as rows, conditions (Reference, Single-cell colony SCC) as columns and the respective protein abundance as colour-coded field. Samples from similar conditions were grouped together and indicated accordingly above the heatmap. The mean protein standard deviation (SD) was calculated for each condition individually by determining the standard deviation of each protein across the 5 respective replicates before calculating the mean over all protein standard deviations. The highest mean protein standard deviation for each cell line is highlighted in bold (TIF 2438 KB)

**Sup. Table 1 Applied chromatographic gradient for peptide separation prior to mass spectrometric measurement for AsPC-1 and PANC-1.** The table describes the proportion of Buffer B (80 % v/v acetonitrile, 0.1 % v/v formic acid) in Buffer A (0.1 % v/v formic acid) over time together with the corresponding flow rate (XLSX 11 KB)

**Sup. Table 2 Applied chromatographic gradient for peptide separation prior to mass spectrometric measurement for MIA PaCa-2.** The table describes the proportion of Buffer B (80 % v/v acetonitrile, 0.1 % v/v formic acid) in Buffer A (0.1 % v/v formic acid) over time together with the corresponding flow rate (XLSX 11 KB)

## Data Availability

All mass spectrometry proteomics datasets generated during and/or analyzed during this study as well as cell authentication certificates are available online in the MassIVE repository (http://massive.ucsd.edu/; dataset identifier: MSV000088904; Reviewer account details: Username: “MSV000088904_reviewer”, Password: “ProteomeClonal”).

## Abbreviations

AcLS: Acid-labile surfactant
ACN: Acetonitrile
Adj.P.Val: Adjusted *p* value
AGC: Automatic gain control
BCA: Bicinchoninic acid
DDA: Data dependent acquisition
DMEM: Dulbecco’s modified Eagle’s medium
DMSO: Dimethylsulfoxid
DPBS: Dulbecco’s phosphate buffered saline
DTT: Dithiothreitol
ECM: Extracellular matrix
EDTA: Ethylenediaminetetraacetic acid
ESI: Electrospray Ionisation
FA: Formic Acid
FACS: Fluorescence activated cell sorting
FC: Fold change
FDR: False discovery rate
GO: Gene ontology
HBSS: Hanks’ balanced salt solution
HEPES: 4-(2-Hydroxyethyl)-1-piperazineethanesulfonic acid
ID: Identification
iRT: Indexed retention time
LC: Liquid chromatography
LFQ: Label-free quantification
LysC: Lysyl endopeptidase
MS: Mass spectrometry
*m*/*z*: Mass-to-charge ratio
PLSDA: Partial least squares discriminant analysis
PRM: Parallel reaction monitoring
Ref.: Reference cell culture
RT: Room temperature
SCC: Single-cell colony
Sp3: Single-pot, solid-phase-enhanced sample-preparation
Stand.: Standard cell culture
TFA: Trifluoroacetic acid
TMT: Tandem mass tag
