# Supplementary figures and images for: Proteome alterations during clonal isolation of established human pancreatic cancer cell lines
Source: Cell Mol Life Sci. 2022 Oct 22;79(11):561. doi: 10.1007/s00018-022-04584-9 (PMC9587952; doi:10.1007/s00018-022-04584-9)

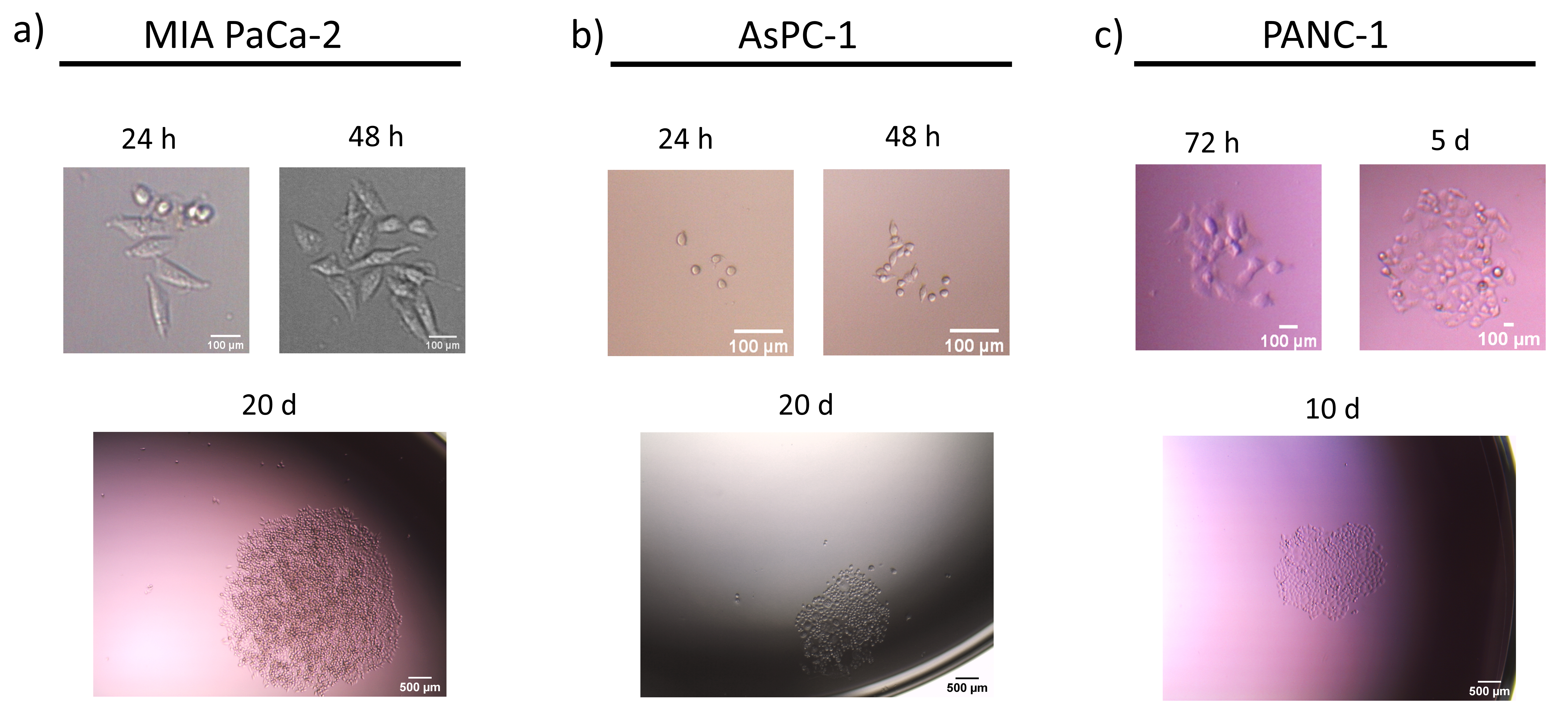

Supplement: Supplementary file 9 — Sup. Fig. 1 Single-Cell Isolation of Human Pancreatic Cell Lines via Limiting Dilution. Human pancreatic cells including a) MIA PaCa-2, b) AsPC-1 and c) PANC-1 were cultured in 96-well flat-bottom plates. Upon limiting dilution, 96-well plates were regularly inspected via light microscopy for emerging single-cell colonies, which could be first observed after 24 hours for MIA PaCa-2 and AsPC-1 and after 72 hours for PANC-1. These single-cell colonies resulted in a spherical colony after 20 days for MIA PaCa-2 and AsPC-1 and after 10 days for PANC-1. The particular size of the scale bar is indicated in each image and has been previously calibrated for each magnification using a Neubauer counting chamber. Contrast and brightness have been adjusted (TIF 15463 KB) [file 18_2022_4584_MOESM9_ESM.tif]

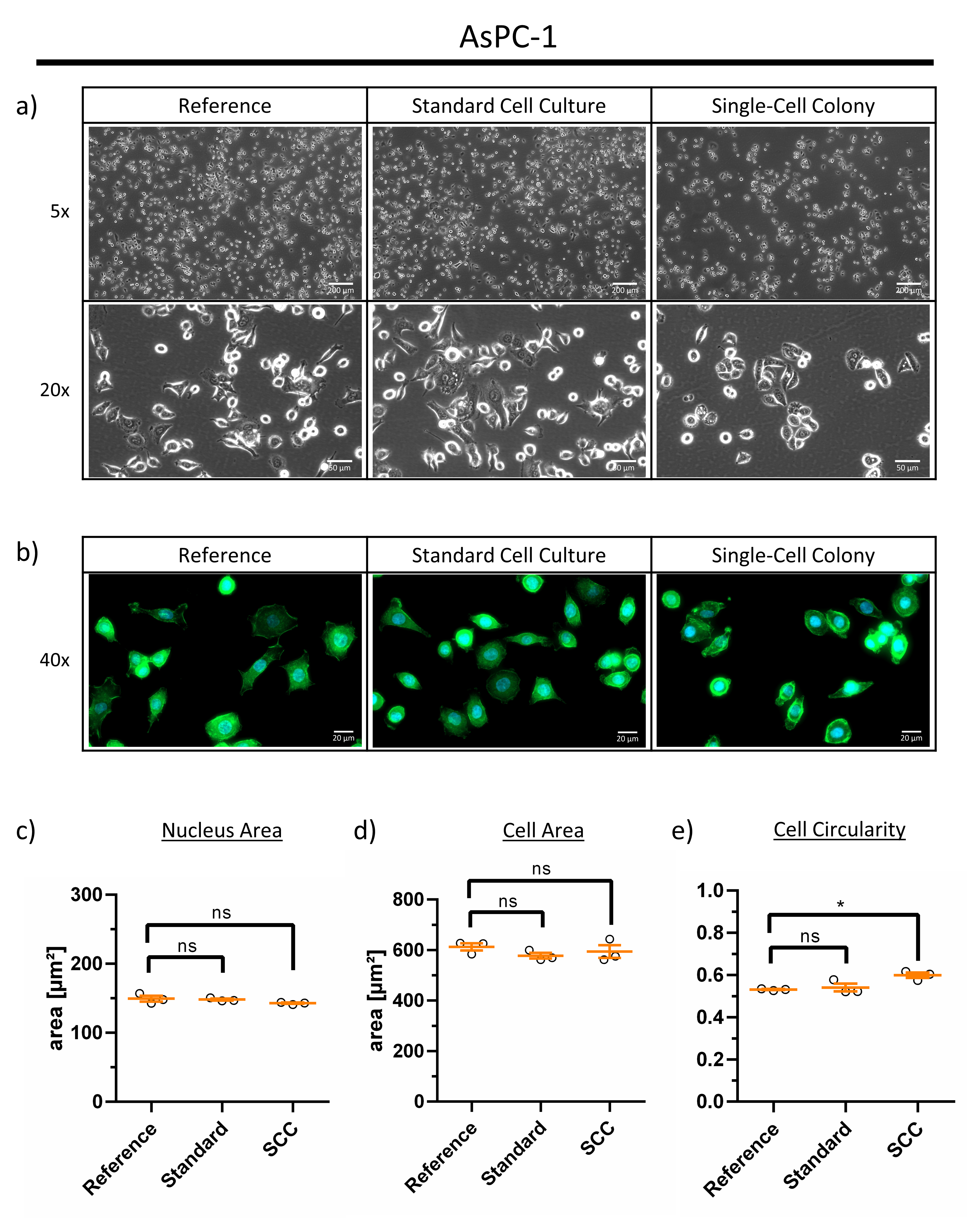

Supplement: Supplementary file 10 — Sup. Fig. 2 Cell Morphology before and after Standard Cell Culture or Clonal Isolation of human pancreatic AsPc-1 cell line. Human pancreatic AsPc-1 reference cells either undergoing standard cell culture or clonal isolation via limiting dilution to obtain single-cell colonies. (a) Cells were cultured in cell culture flasks and imaged via phase contrast microscopy (5x and 20x magnification). (b) Cells were fluorescence stained for F-actin by fluorophore labelled Phalloidin (green) and dsDNA by Hoechst 33342 (blue). Maximum intensity projection of 40x z-stack images is shown. (c–e) Morphometric parameters of individual cells were analysed based on Phalloidin and Hoechst 33342 fluorescence staining. Three independent replicates and at least 388 nuclei (c) or 100 cells (d,e) per condition and replicate were analysed. Scatter plot dots represent mean values per replicate (error bars show mean and S.E.M., * – p<0.05, ns – non significant) (TIF 7076 KB) [file 18_2022_4584_MOESM10_ESM.tif]

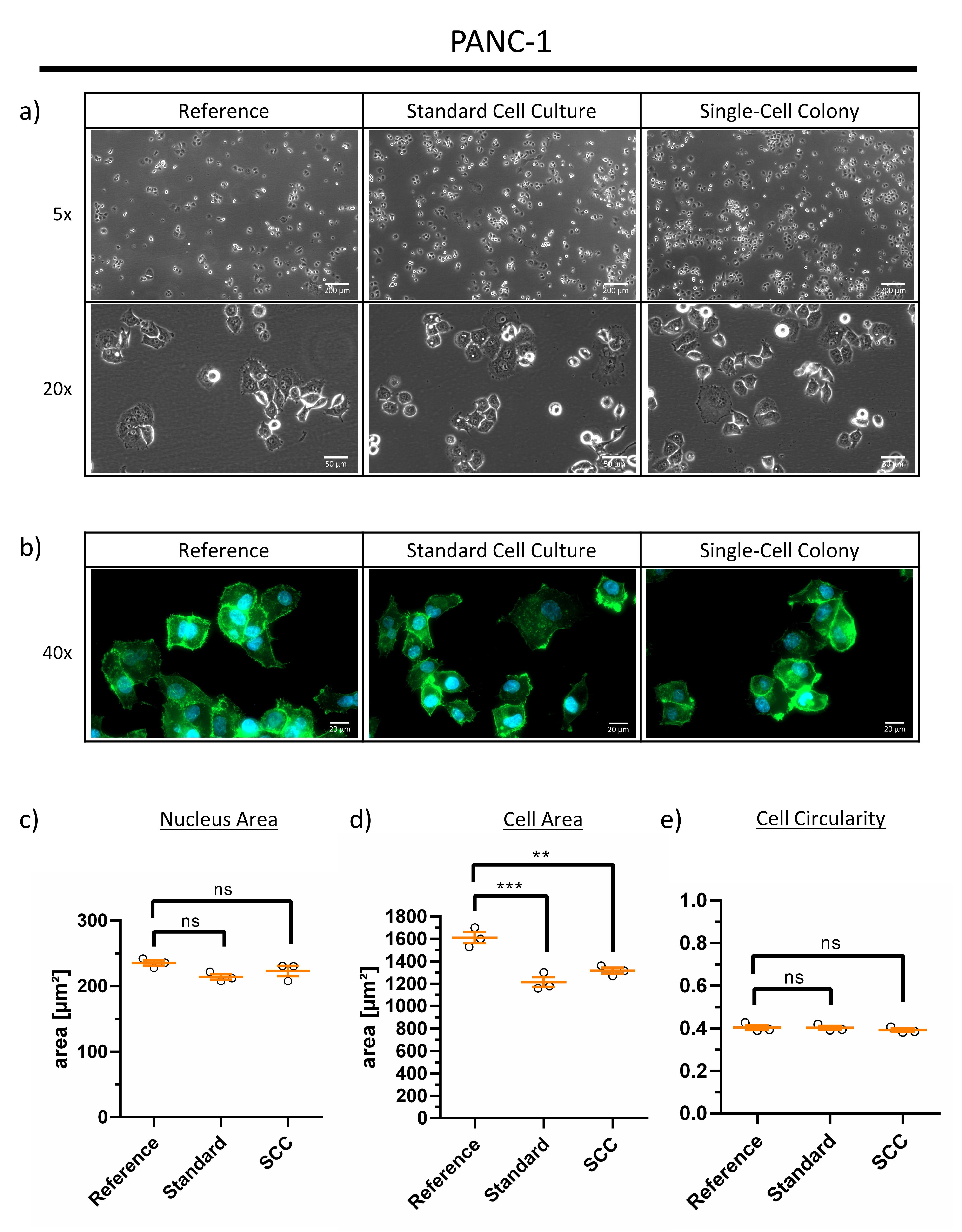

Supplement: Supplementary file 11 — Sup. Fig. 3 Cell Morphology before and after Standard Cell Culture or Clonal Isolation of human pancreatic PANC-1 cell line. Human pancreatic PANC-1 reference cells either undergoing standard cell culture or clonal isolation via limiting dilution to obtain single-cell colonies. (a) Cells were cultured in cell culture flasks and imaged via phase contrast microscopy (5x and 20x magnification). (b) Cells were fluorescence stained for F-actin by fluorophore labelled Phalloidin (green) and dsDNA by Hoechst 33342 (blue). Maximum intensity projection of 40x z-stack images is shown. (c–e) Morphometric parameters of individual cells were analysed based on Phalloidin and Hoechst 33342 fluorescence staining. Three independent replicates and at least 272 nuclei (c) or 100 cells (d,e) per condition and replicate were analysed. Scatter plot dots represent mean values per replicate (error bars show mean and S.E.M., ** – p<0.01, *** – p<0.001, ns – non significant) (TIF 7285 KB) [file 18_2022_4584_MOESM11_ESM.tif]

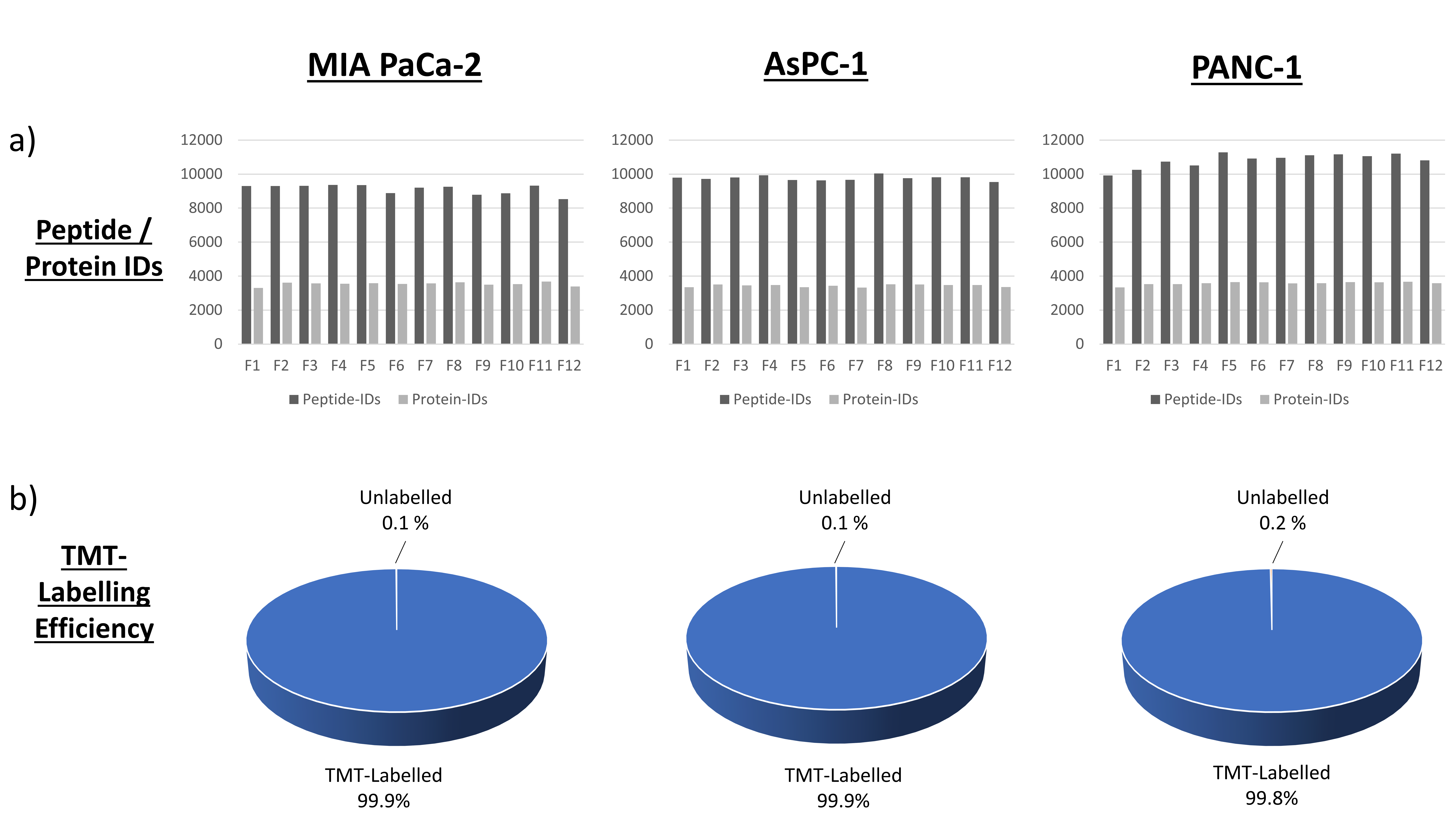

Supplement: Supplementary file 12 — Sup. Fig. 4 Proteomic Data Characteristics including Number of Identified Peptides and Proteins and Labelling Efficiency. For peptide and protein identification, acquired LC-MS/MS data was searched against a human database using MaxQuant with 1 % false discovery rate (FDR). (a) Resulting numbers of identified peptides and proteins are shown for each fraction (F1–F12) and cell line (MIA PaCa-2, AsPC-1, PANC-1). (b) The efficiency of peptide-level TMT-labelling was evaluated by calculating the intensity-ratio of N-terminally labelled, tryptic peptides against all identified tryptic peptides (TIF 889 KB) [file 18_2022_4584_MOESM12_ESM.tif]

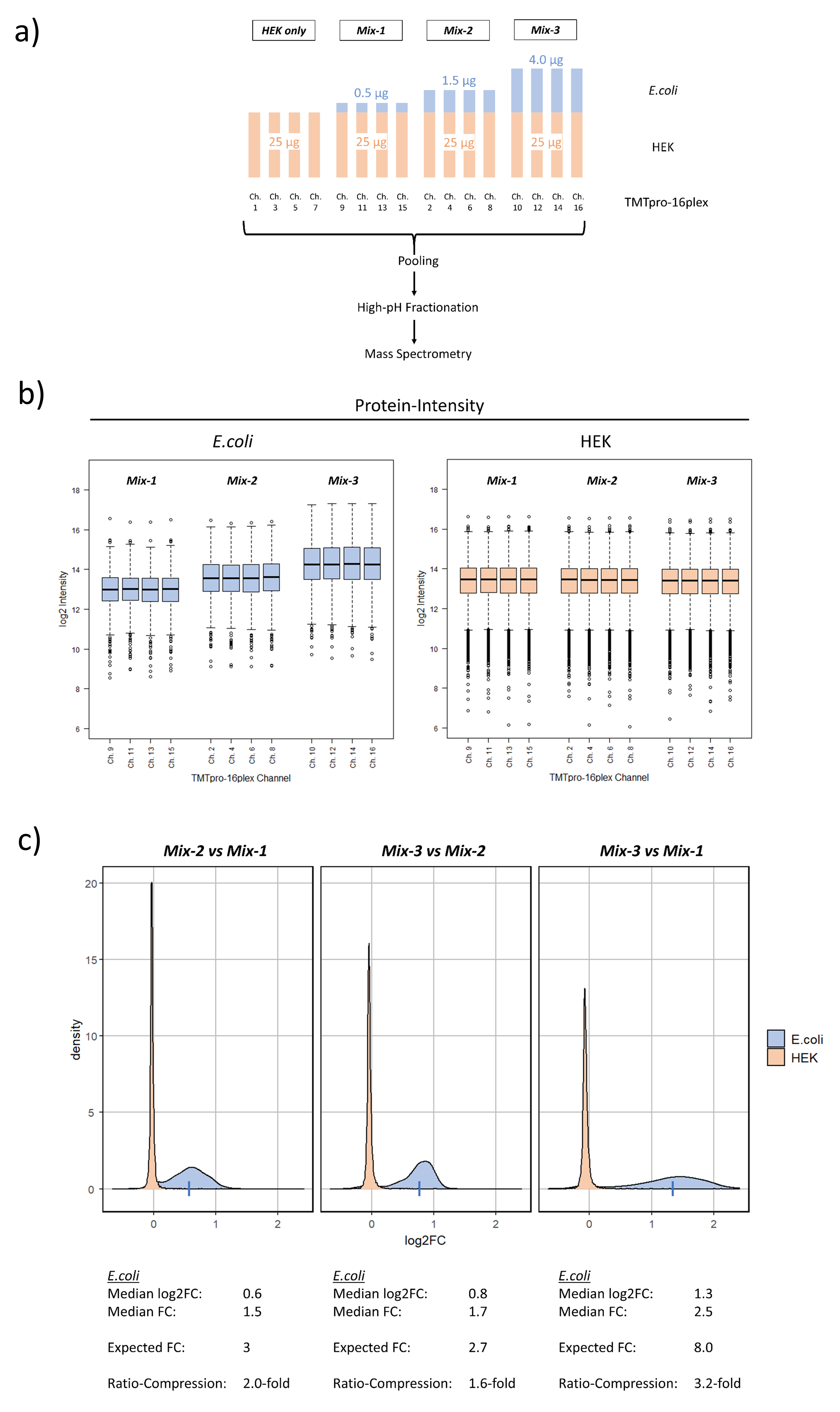

Supplement: Supplementary file 13 — Sup. Fig. 5 Applicability Validation of TMTpro-16plex using a HEK-E.Coli Benchmark Dataset. (a) The TMTpro-16plex benchmark dataset was created by adding different amounts of E.coli peptides (0.5 µg, 1.5 µg, 4 µg) to a consistent amount of human HEK peptides (20 µg), resulting in one “Hek only” condition and three mixtures of know peptide mass ratios (E.coli:HEK 1:50, 1:17, 1:6) with four replicates each. After isobaric labelling with tandem mass tag (TMTpro-16plex), all samples were pooled and offline fractionated using high-pH reversed phase chromatography. Resulting fractions were subjected to mass spectrometric analysis operated in data-dependent acquisition mode (DDA). Database search of resulting raw data was performed using MaxQuant with subsequent assignment to E.Coli and HEK proteins. (b) Protein intensities after median normalization with MSstatsTMT are illustrated in log2 scale for each TMT channel of the mixtures in the E.coli and the HEK fraction as boxplots, representing corresponding protein abundance. Lower and upper box boundaries represent the 25th and 75th percentiles, line inside the box represent median, upper and lower error lines represent 1.5 times the interquartile range and circles represent data points exceeding that error range. (c) Differential expression analysis between mixtures was performed using a pairwise multigroup limma approach. The distribution of resulting fold changes for each comparison (“Mix-2 vs Mix-1”, “Mix-3 vs Mix-2”, “Mix-3 vs Mix-2”) are illustrated in log2 scale as density plots. For the E.coli fraction, the observed median log2 fold change and the therefrom derived delogarithmized median fold change ratio are stated below each comparison plot. Together with the expected fold change based on the known mass ratio of added E.coli peptides (3:1, 2.7:1, 8:1), the respective ratio compression factor could be calculated (TIF 2610 KB) [file 18_2022_4584_MOESM13_ESM.tif]

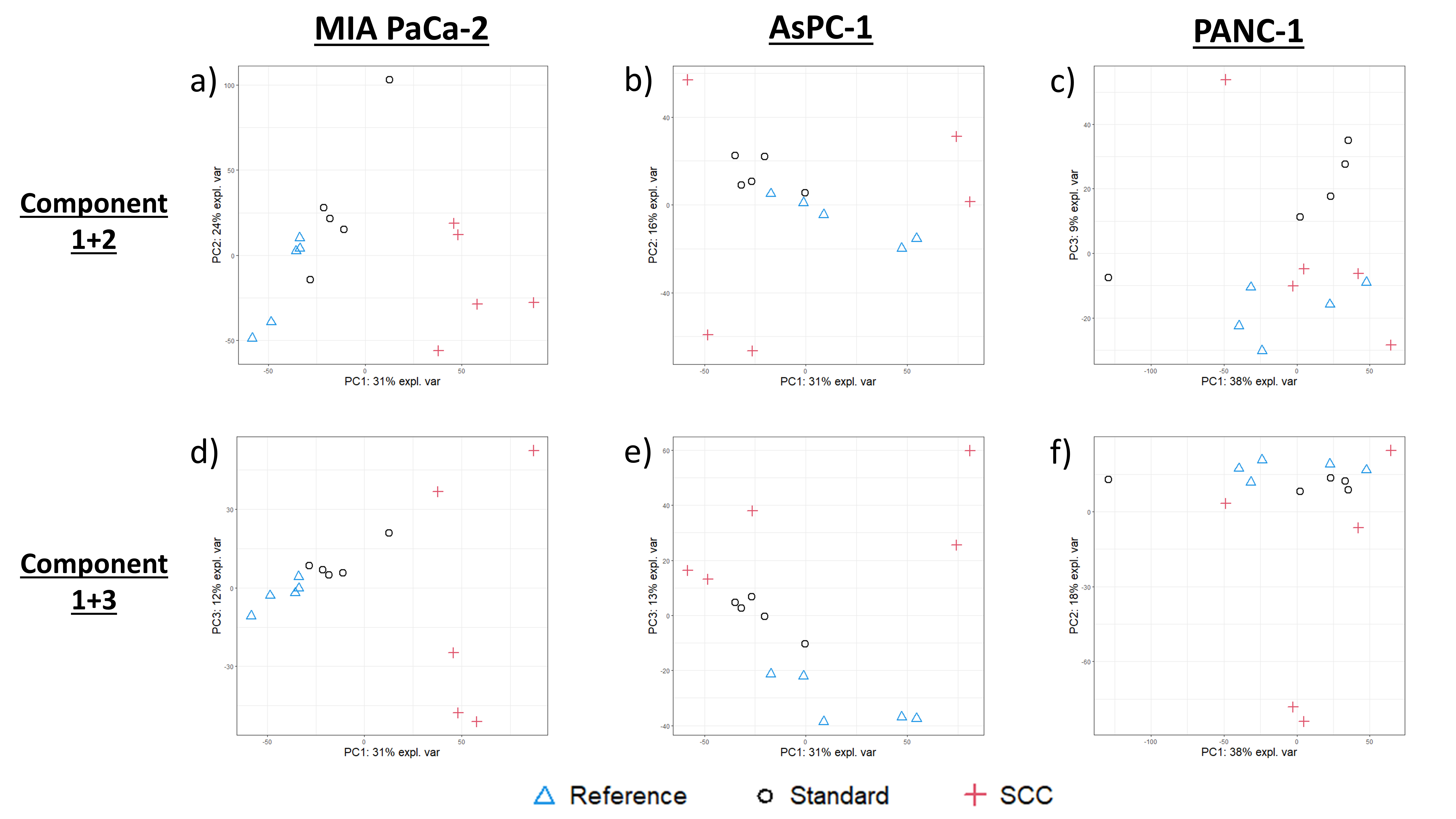

Supplement: Supplementary file 14 — Sup. Fig. 6 Principal Component Analysis (PCA). Protein profiles of each sample were submitted to PCA analysis. For the analysis either components 1 and 2 (a,b,c) or components 1 and 3 (d,e,f) were considered, where x- and y-axis represent the percentage of explained variance of the respective component (TIF 3560 KB) [file 18_2022_4584_MOESM14_ESM.tif]

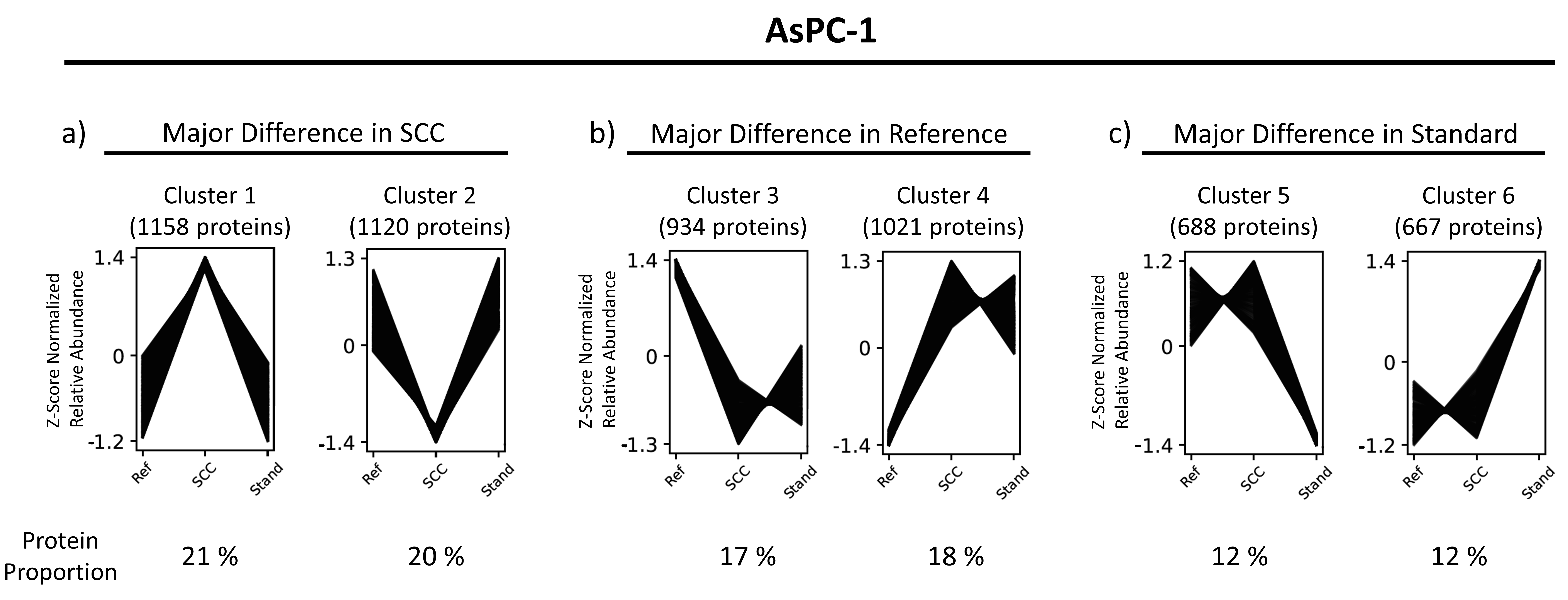

Supplement: Supplementary file 15 — Sup. Fig. 7 Co-Abundance Cluster Analysis of Identified Proteins from AsPC-1. Depending on the measured intensities in different conditions (Reference (Ref), Single-cell colony (SCC), Standard cell culture (Stand)), the identified proteins were assigned to different co-abundance cluster with a confidence interval of 95 %. Cluster assignment was performed using the Clust algorithm. Each line represents an individual protein, while the y-axis illustrates the relative abundance change after Z-score normalization. Co-Abundance clusters were sorted into three groups (a,b,c), depending on the condition showing the major difference. The number of assigned proteins per cluster is shown above each graph and the corresponding proportion of the total number of assigned proteins is shown below each graph (TIF 1498 KB) [file 18_2022_4584_MOESM15_ESM.tif]

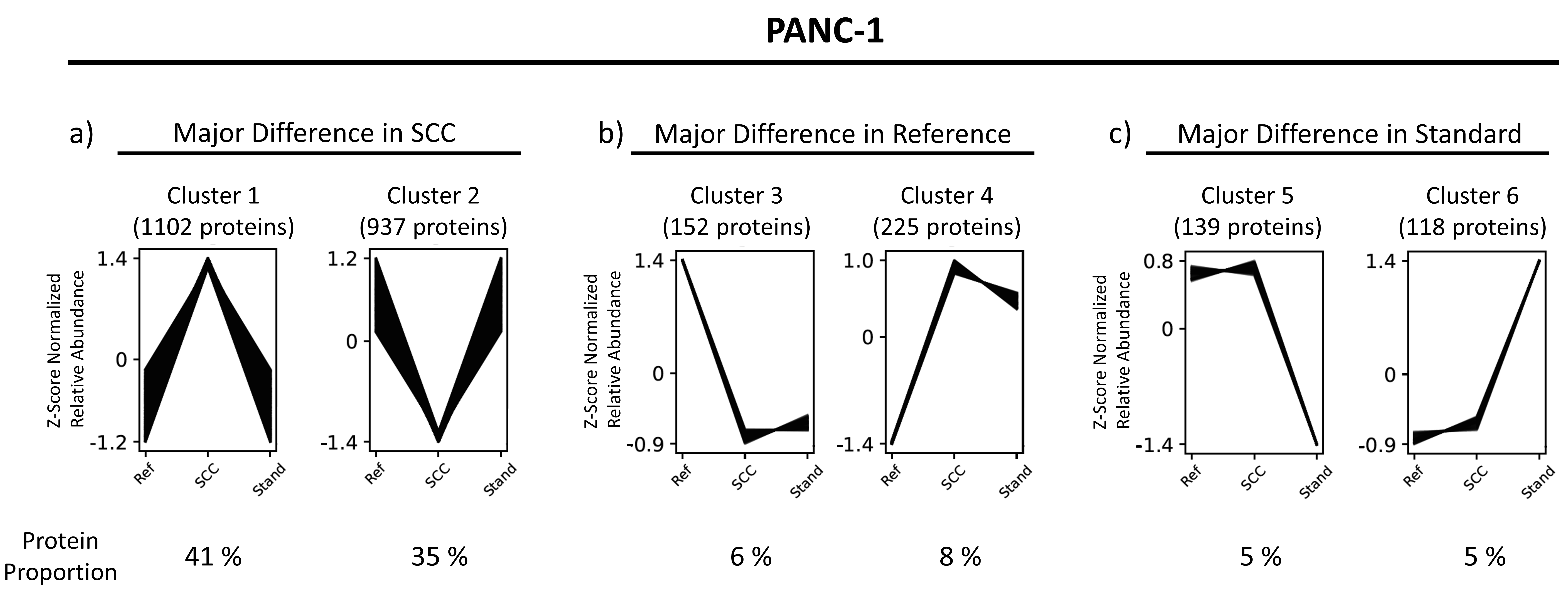

Supplement: Supplementary file 16 — Sup. Fig. 8 Co-Abundance Cluster Analysis of Identified Proteins from PANC-1. Depending on the measured intensities in different conditions (Reference (Ref), Single-cell colony (SCC), Standard cell culture (Stand)), the identified proteins were assigned to different co-abundance cluster with a confidence interval of 95 %. Cluster assignment was performed using the Clust algorithm. Each line represents an individual protein, while the y-axis illustrates the relative abundance change after Z-score normalization. Co-Abundance clusters were sorted into three groups (a,b,c), depending on the condition showing the major difference. The number of assigned proteins per cluster is shown above each graph and the corresponding proportion of the total number of assigned proteins is shown below each graph (TIF 1455 KB) [file 18_2022_4584_MOESM16_ESM.tif]

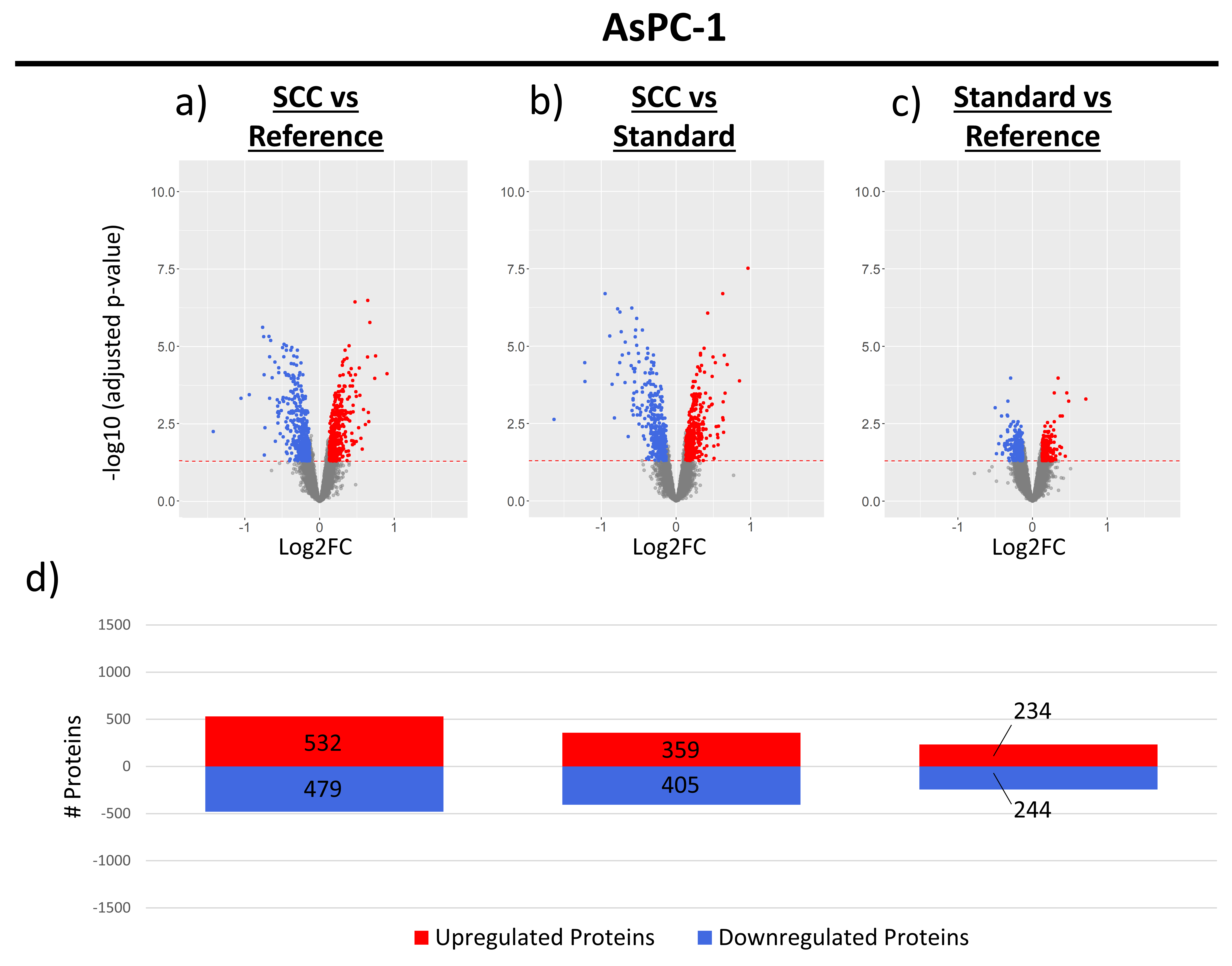

Supplement: Supplementary file 17 — Sup. Fig. 9 Differential Expression Analysis for AsPC-1 undergoing Clonal Isolation or Standard Cell Culture. For differential expression analysis, a pairwise multigroup limma approach was used to compare the conditions a) “Single-Cell Culture (SCC) vs Reference”, b) “Single-Cell Culture (SCC) vs Standard Cell Culture” and c) “Standard Cell Culture vs Reference” while results were illustrated as volcano plots. The log2 fold changes (log2FC) are plotted on the x-axis and corresponding adjusted p-values in -log10 scale are shown on the y-axis. The applied adjusted p-value cut-off was set to 0.05 (1.3 in -log10 scale, depicted as dashed horizonal line), while the log2FC cut-off was set to +/- 0.13 corresponding to 10 % FC. Each plot highlights significantly up- (red) or down-regulated (blue) proteins. Hereby, a log2FC > 0 corresponds to an upregulation in the first-mentioned condition. (d) Numbers of significantly up- and down-regulated proteins for each comparison are illustrated as bar chart (TIF 1215 KB) [file 18_2022_4584_MOESM17_ESM.tif]

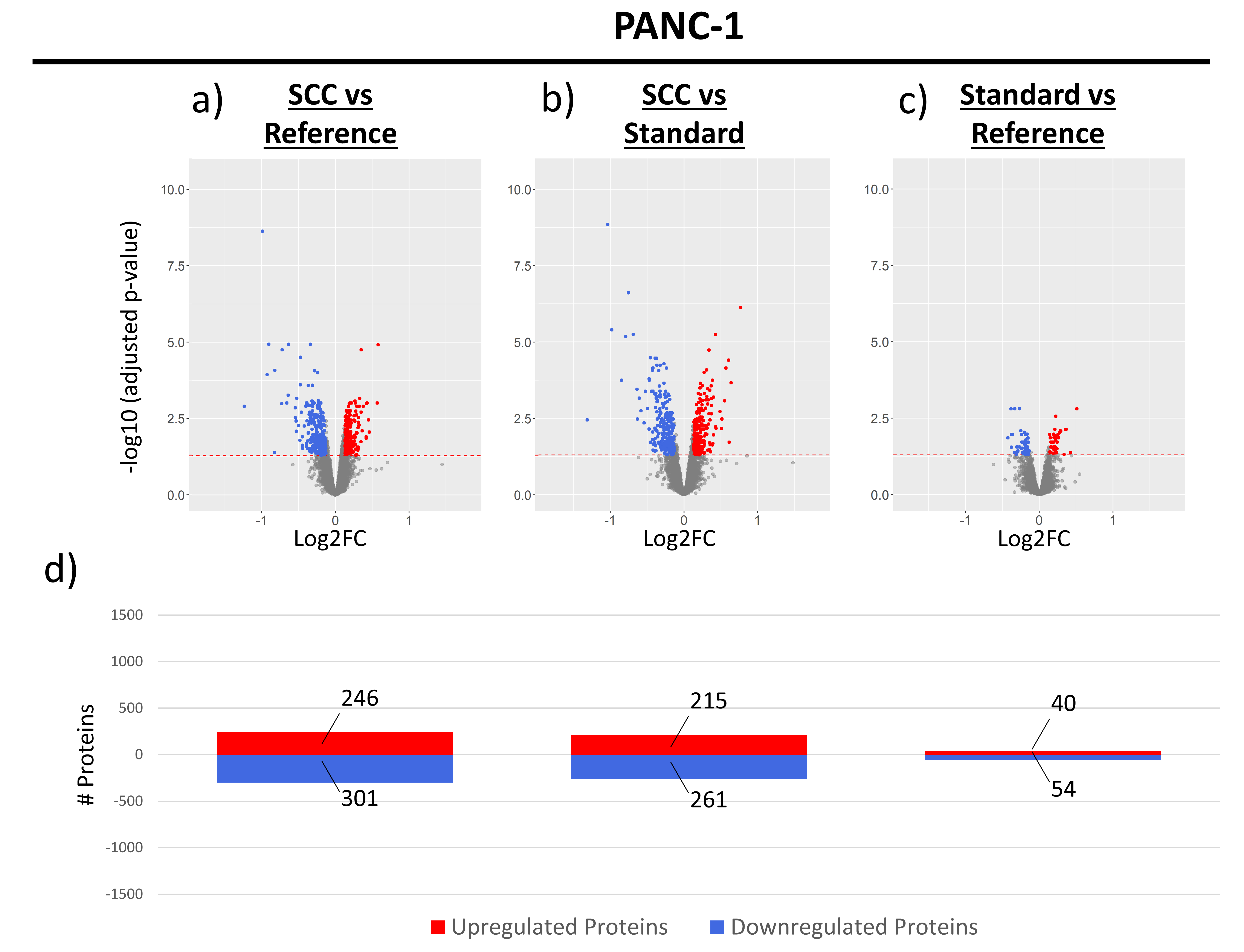

Supplement: Supplementary file 18 — Sup. Fig. 10 Differential Expression Analysis for PANC-1 undergoing Clonal Isolation or Standard Cell Culture. For differential expression analysis, a pairwise multigroup limma approach was used to compare the conditions a) “Single-Cell Culture (SCC) vs Reference”, b) “Single-Cell Culture (SCC) vs Standard Cell Culture” and c) “Standard Cell Culture vs Reference” while results were illustrated as volcano plots. The log2 fold changes (log2FC) are plotted on the x-axis and corresponding adjusted p-values in -log10 scale are shown on the y-axis. The applied adjusted p-value cut-off was set to 0.05 (1.3 in -log10 scale, depicted as dashed horizonal line), while the log2FC cut-off was set to +/- 0.13 corresponding to 10 % FC. Each plot highlights significantly up- (red) or down-regulated (blue) proteins. Hereby, a log2FC > 0 corresponds to an upregulation in the first-mentioned condition. (d) Numbers of significantly up- and down-regulated proteins for each comparison are illustrated as bar chart (TIF 1152 KB) [file 18_2022_4584_MOESM18_ESM.tif]

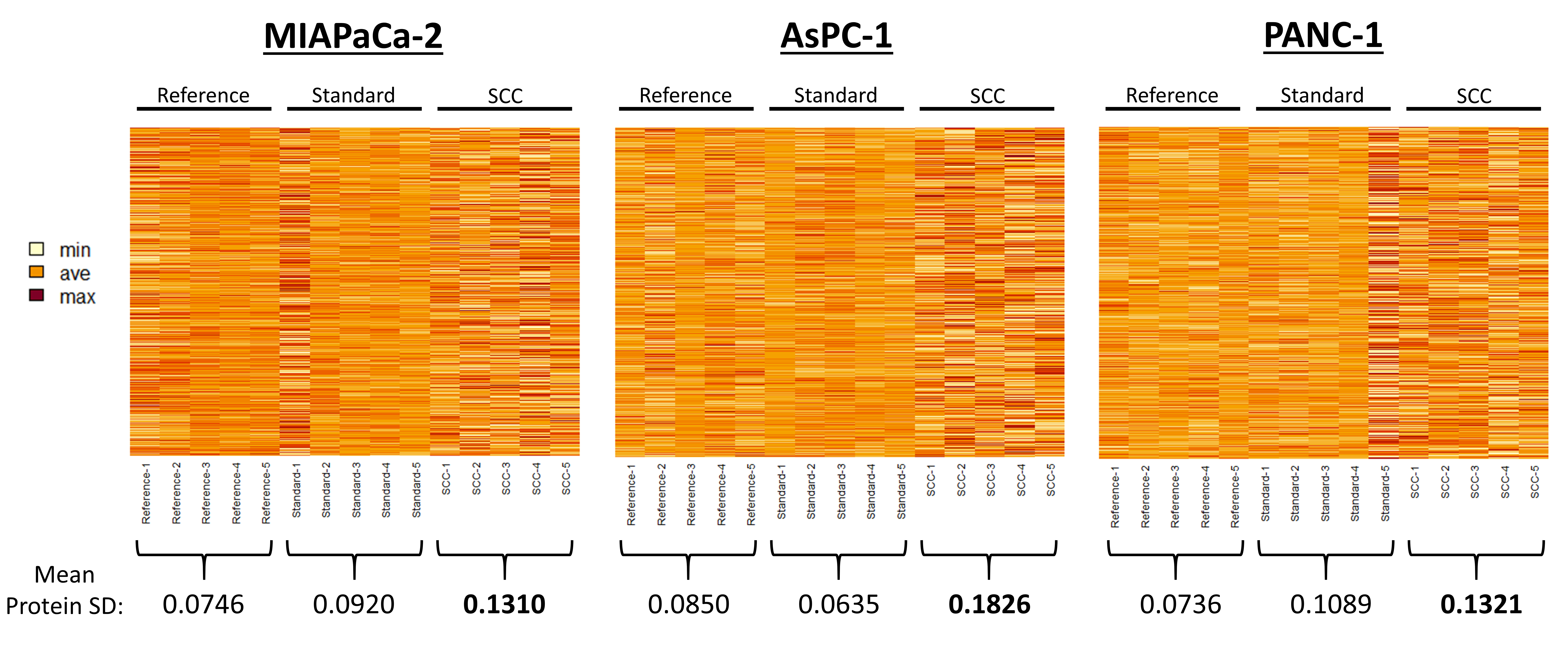

Supplement: Supplementary file 19 — Sup. Fig. 11 Heatmap Representation of Cell Line Specific Protein Expression Profiles and Mean Protein Standard Deviation per Condition. Acquired proteomic data for MIA PaCa-2, AsPC-1 and PANC-1 were visualised via heatmap representing proteins as rows, conditions (Reference, Standard, Single-cell colony SCC) as columns and the respective protein abundance as colour-coded field. Samples from similar conditions were grouped together and indicated accordingly above the heatmap. The mean protein standard deviation (SD) was calculated for each condition individually by determining the standard deviation of each protein across the 5 respective replicates before calculating the mean over all protein standard deviations. The highest mean protein standard deviation for each cell line is highlighted in bold (TIF 3438 KB) [file 18_2022_4584_MOESM19_ESM.tif]

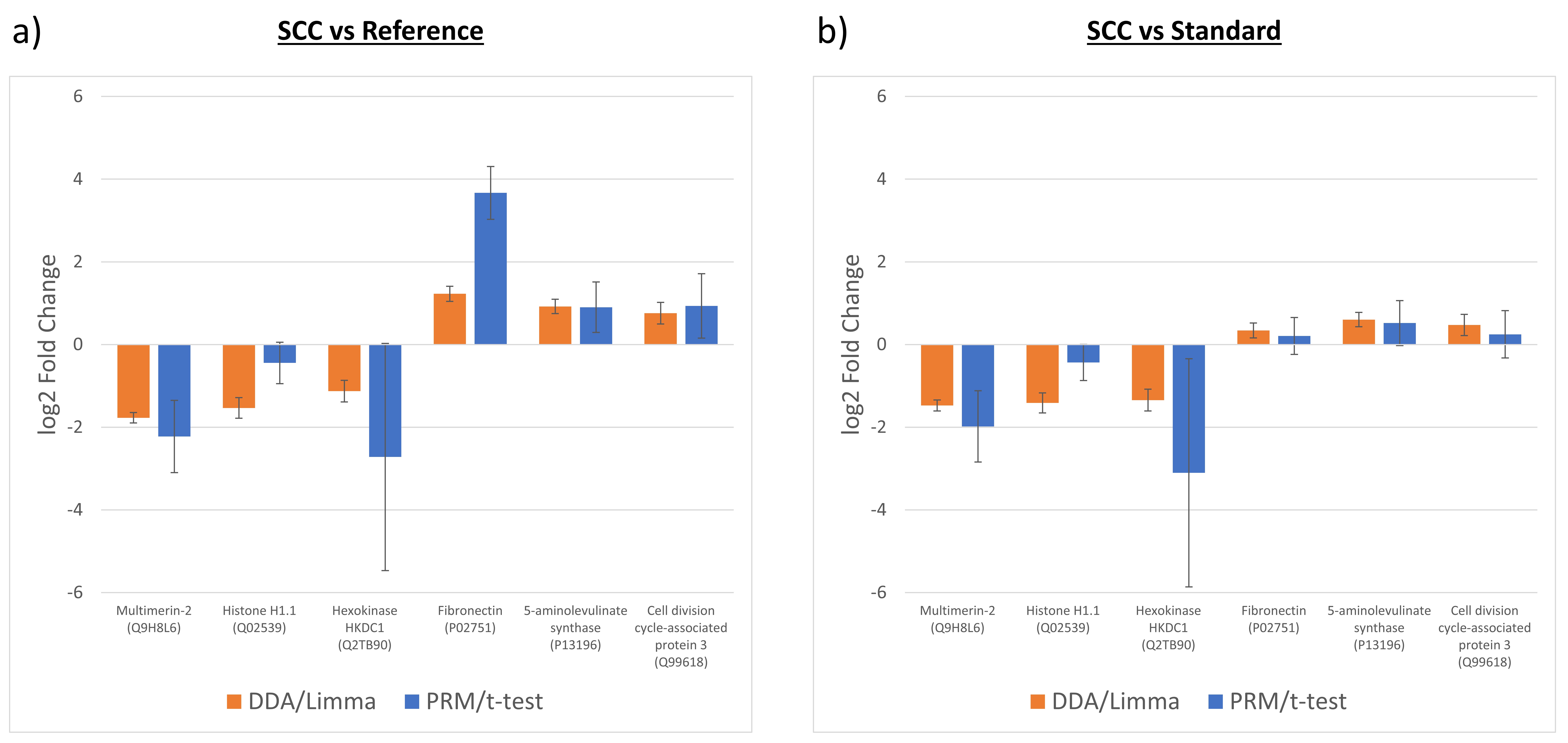

Supplement: Supplementary file 20 — Sup. Fig. 12 Comparison of log2 fold changes from data-dependent acquisition (DDA) mode and targeted parallel reaction monitoring (PRM) mode in MIA PaCa-2. Labelled or unlabelled peptide samples were analysed in DDA- or PRM-mode and subjected to pairwise comparisons using multigroup limma or two-sample t-test, respectively. Resulting log2 fold changes for the DDA/Limma- (orange) and PRM/t-test-approach are shown as bar plots for 6 exemplary proteins (with corresponding Uniprot-IDs) for the comparisons a) “Single-Cell Culture (SCC) vs Reference” and b) “Single-Cell Culture (SCC) vs Standard Cell Culture”. Error bars correspond to the 95 % confidence intervals (TIF 618 KB) [file 18_2022_4584_MOESM20_ESM.tif]

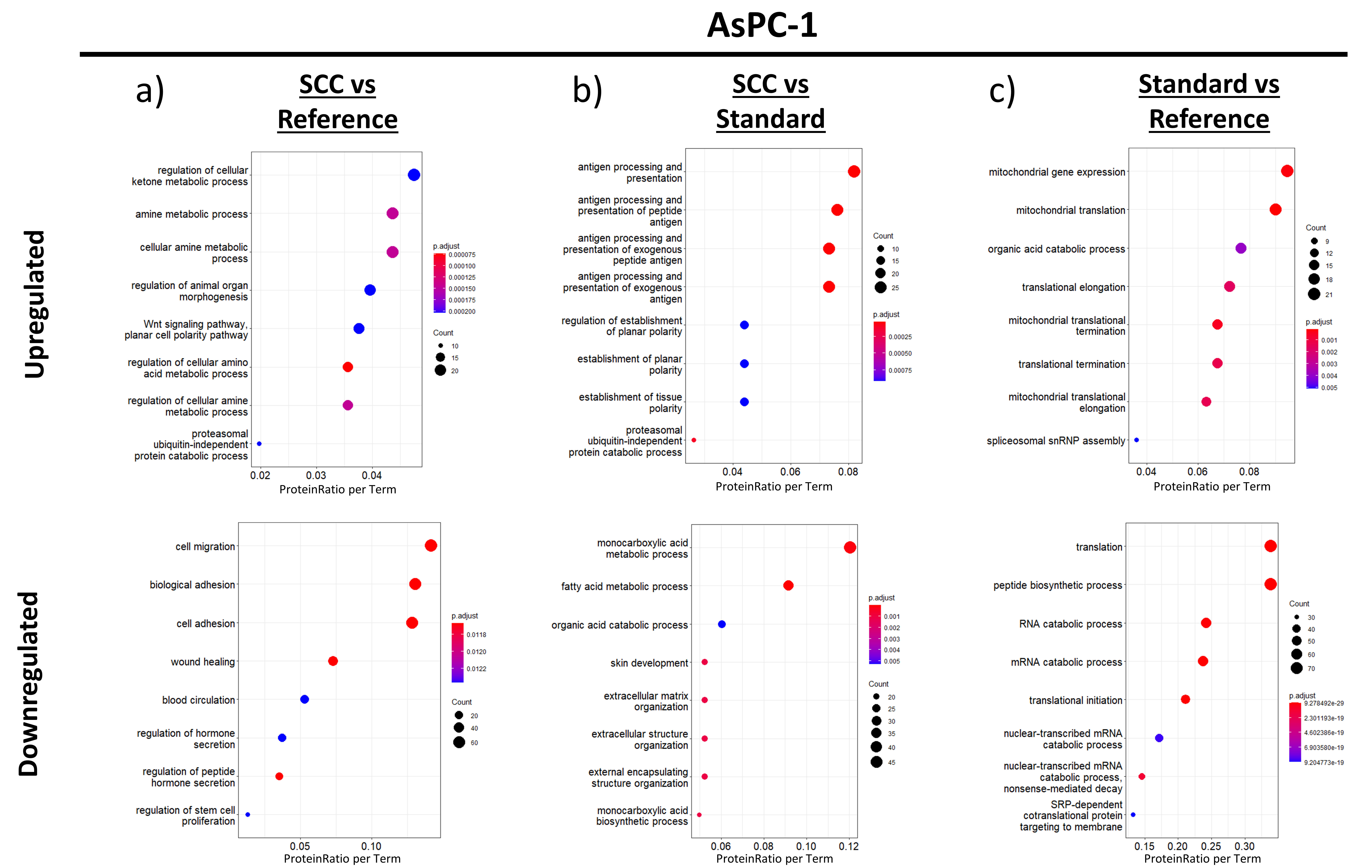

Supplement: Supplementary file 21 — Sup. Fig. 13 Gene Ontology Enrichment Analysis (Biological Process) of differentially expressed proteins in AsPC-1. Gene Ontology (GO) enrichment analysis of differentially expressed proteins was retrieved using ClusterProfiler in R resulting in dot plots for the comparisons a) “Single-Cell Culture (SCC) vs Reference”, b) “Single-Cell Culture (SCC) vs Standard Cell Culture” and c) “Standard Cell Culture vs Reference”. The y-axis represents the GO terms, while the x-axis illustrates the proportion of differentially up- or downregulated proteins that are annotated with the respectively shown GO term (ProteinRatio per Term). The colour of the dots corresponds to the adjusted p-value of the GO-enrichment and the size of the dots is proportional to the absolute number of differentially expressed proteins enriched in the respective GO-term. The 8 most significantly enriched (adjusted p-value < 0.05) GO terms in the biological process branch are separately illustrated for up- and downregulated proteins for each comparison (TIF 6488 KB) [file 18_2022_4584_MOESM21_ESM.tif]

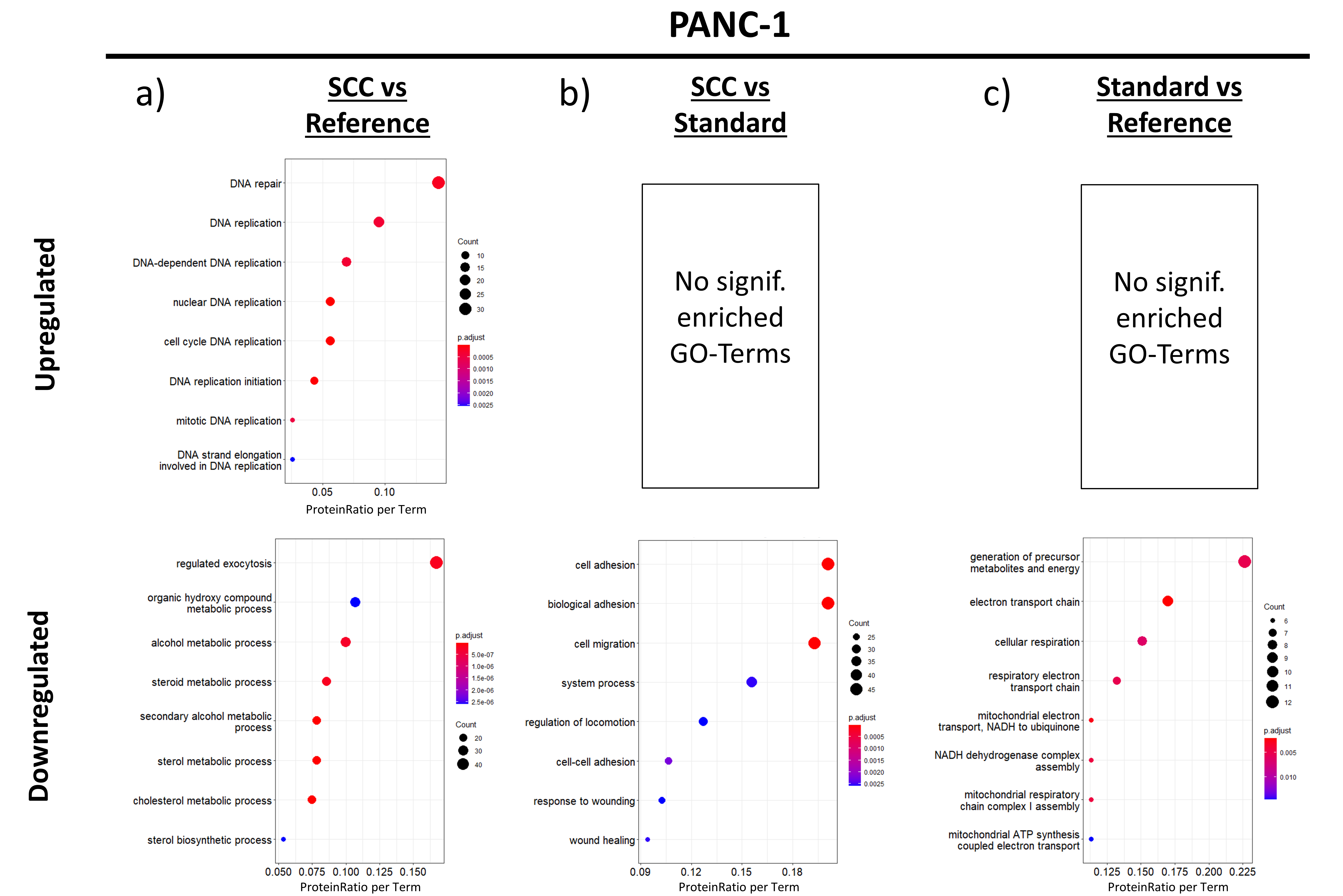

Supplement: Supplementary file 22 — Sup. Fig. 14 Gene Ontology Enrichment Analysis (Biological Process) of differentially expressed proteins in PANC-1. Gene Ontology (GO) enrichment analysis of differentially expressed proteins was retrieved using ClusterProfiler in R resulting in dot plots for the comparisons a) “Single-Cell Culture (SCC) vs Reference”, b) “Single-Cell Culture (SCC) vs Standard Cell Culture” and c) “Standard Cell Culture vs Reference”. The y-axis represents the GO terms, while the x-axis illustrates the proportion of differentially up- or downregulated proteins that are annotated with the respectively shown GO term (ProteinRatio per Term). The colour of the dots corresponds to the adjusted p-value of the GO-enrichment and the size of the dots is proportional to the absolute number of differentially expressed proteins enriched in the respective GO-term. The 8 most significantly enriched (adjusted p-value < 0.05) GO terms in the biological process branch are separately illustrated for up- and downregulated proteins of each comparison (TIF 4045 KB) [file 18_2022_4584_MOESM22_ESM.tif]

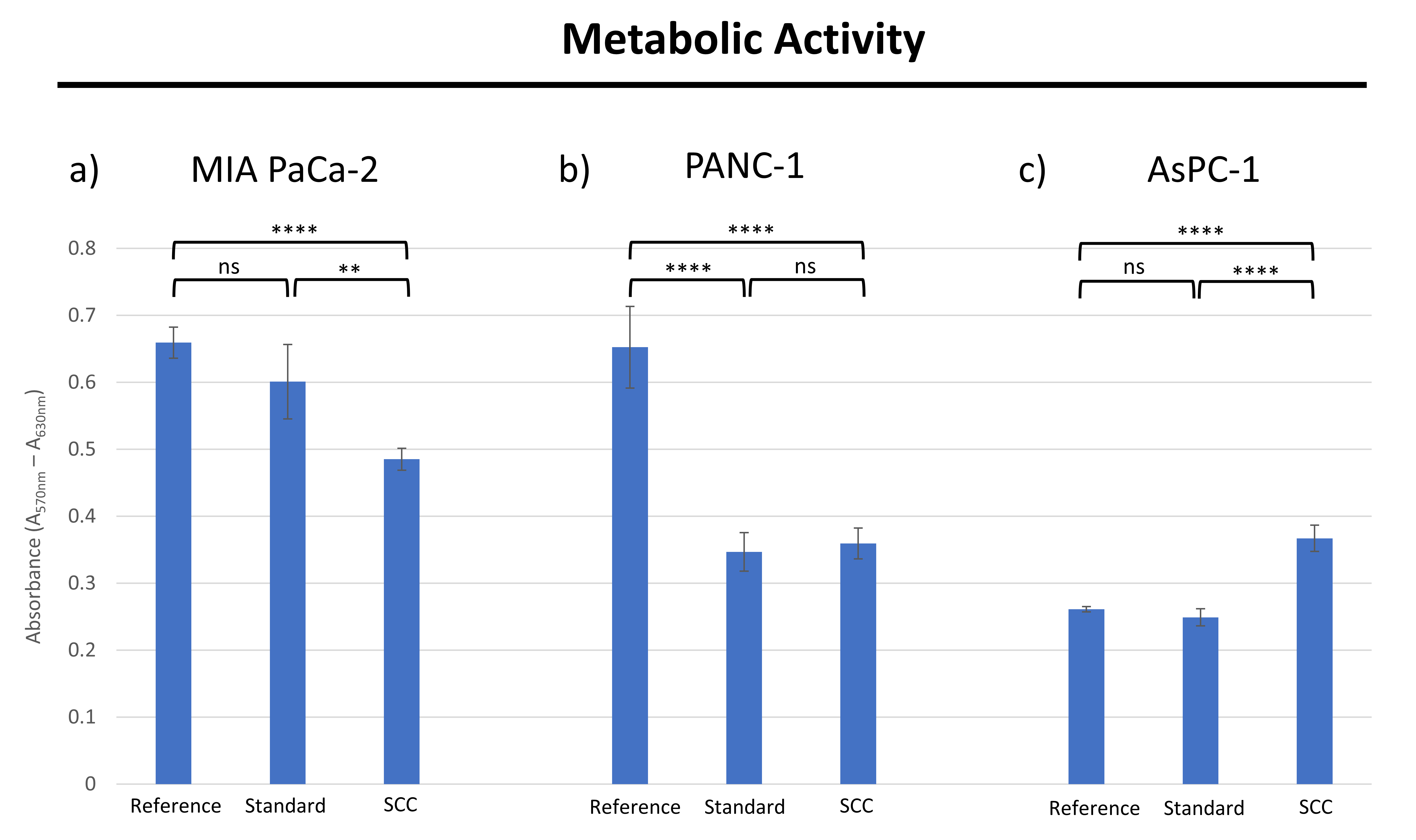

Supplement: Supplementary file 23 — Sup. Fig. 15 Evaluation of cellular metabolic activity by colorimetric MTT-based cell culture assay. Human pancreatic reference cells (MIA PaCa-2, PANC-1, AsPC-1) either undergo standard cell culture (Standard) or clonal isolation via limiting dilution (SCC). For each condition, 5,000 cells/well of the respective cell line were seeded and cultured for 48 hours before analysing metabolic activity with colorimetric MTT-based assay at 570 nm (reference wavelength 630 nm). Each condition was performed with n=7 independent replicates. The bar chart shows the mean of blank-corrected absorbance values and corresponding standard deviation as error bars. One-way ANOVA with Tukey´s multiple comparisons test was used. Statistical significance was defined as ** - p<0.01, **** - p<0.0001, ns – non significant (TIF 412 KB) [file 18_2022_4584_MOESM23_ESM.tif]

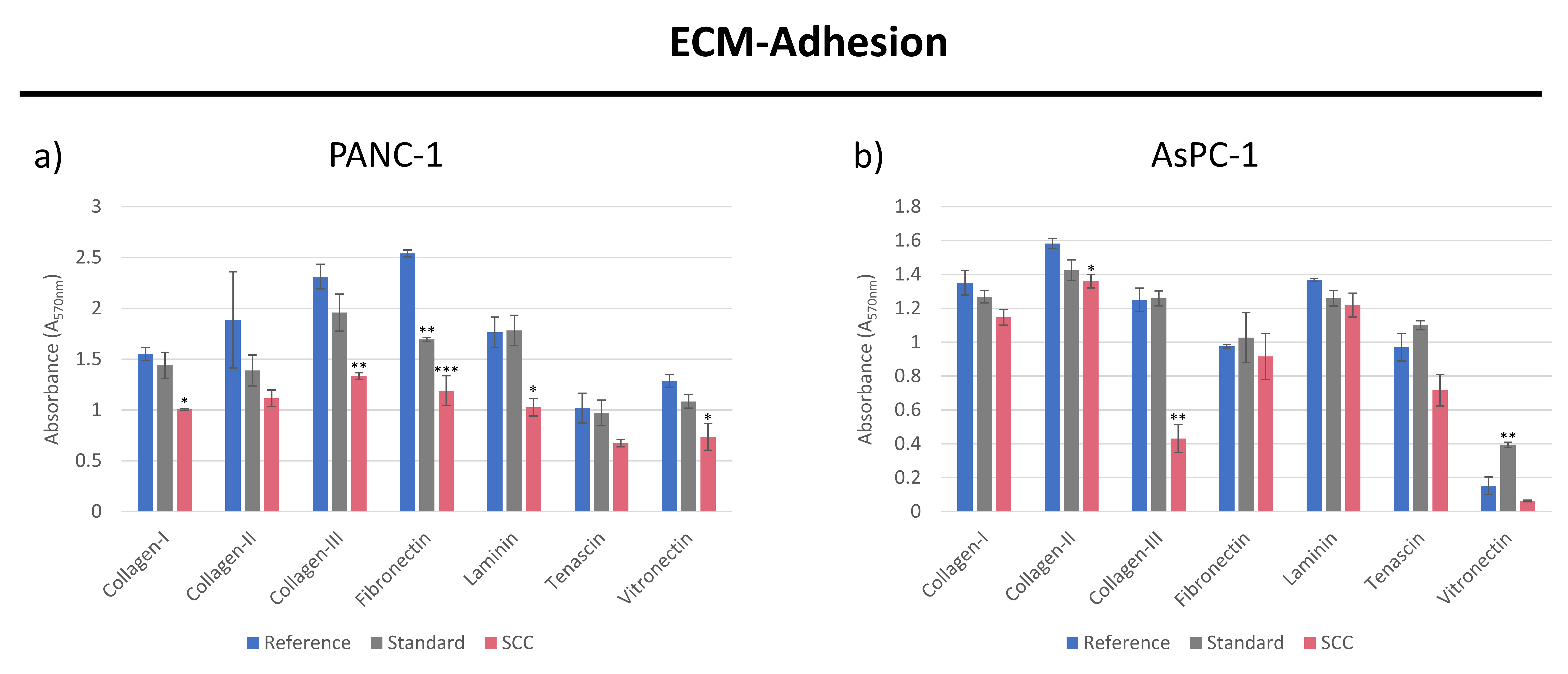

Supplement: Supplementary file 24 — Sup. Fig. 16 Evaluation of cell adhesion capability by colorimetric extracellular matrix (ECM) adhesion assay. Human pancreatic reference cells (PANC-1, AsPC-1) either undergo standard cell culture (Standard) or clonal isolation via limiting dilution (SCC). For each condition, 150,000 cells of the respective cell line were added to each precoated well and incubated for 2 hours before analysing cell adhesion capability by colorimetric ECM adhesion assay at 570 nm. Each condition was performed in duplicates. The bar chart shows the mean of blank-corrected absorbance values and corresponding standard deviation as error bars. One-way ANOVA with Dunnett´s multiple comparisons test was used. Statistical significance to the reference was defined as * - p<0.05, ** - p<0.01, *** - p<0.001, while unlabeled bars correspond to non-significant changes (TIF 557 KB) [file 18_2022_4584_MOESM24_ESM.tif]

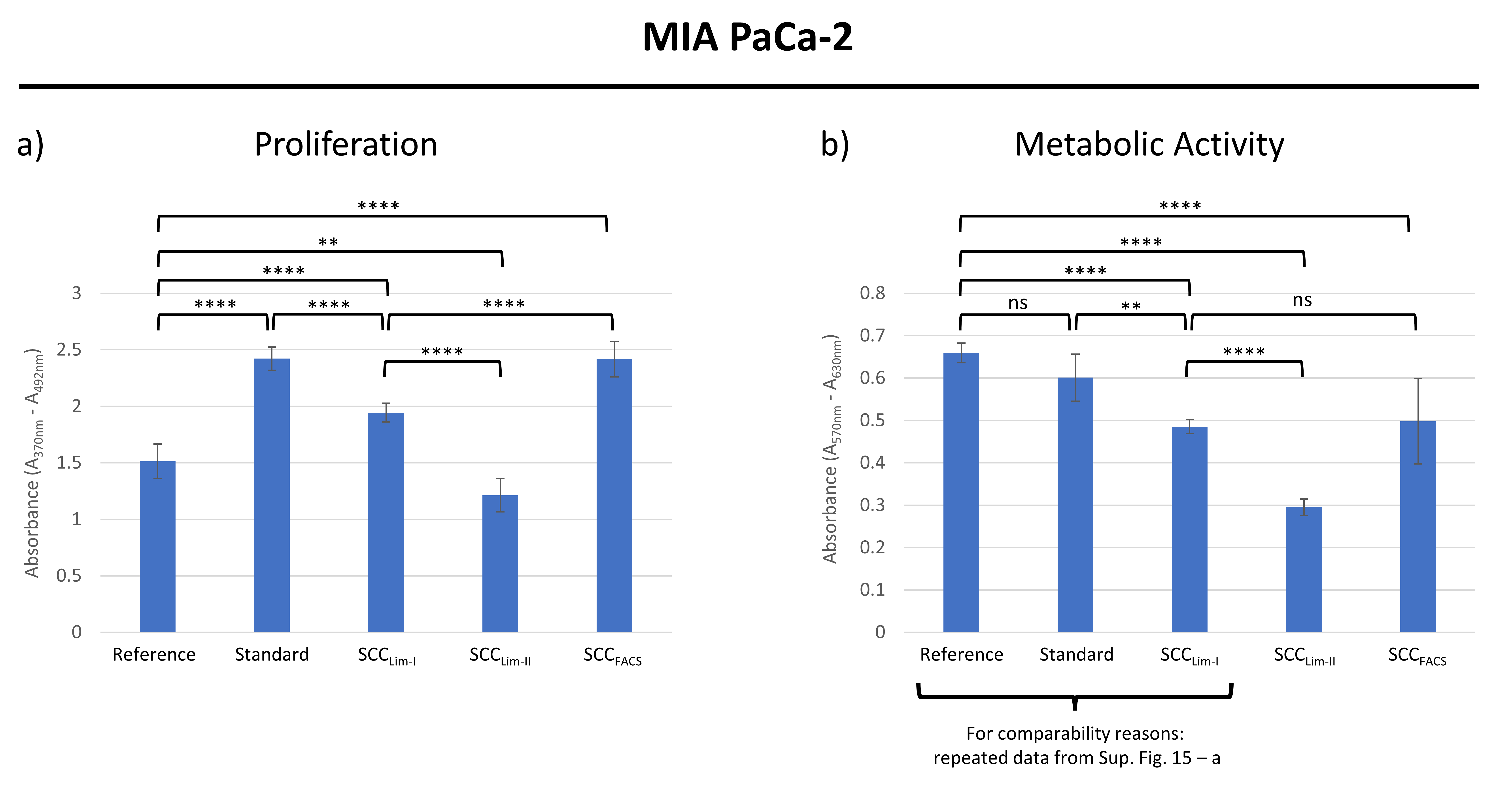

Supplement: Supplementary file 25 — Sup. Fig. 17 Evaluation of proliferation and metabolic activity of MIA PaCa-2 cells. Human MIA PaCa-2 reference cells either undergo standard cell culture (Standard), clonal isolation via a single round of limiting dilution (SCCLim-I), two successive rounds of limiting dilution (SCCLim-II) or clonal isolation via FACS-assisted single-cell isolation (SCCFACS). For each condition, 5,000 cells/well were seeded and cultured for 48 hours before either analysing proliferation with colorimetric BrdU-incorporation ELISA-assay at 370 nm (492 nm reference wavelength) or metabolic activity with colorimetric MTT-based assay at 570 nm (reference wavelength 630 nm). Each condition was performed with n=7 independent replicates. The bar chart shows the mean of blank-corrected absorbance values and corresponding standard deviation as error bars. Metabolic activity data for the conditions “Reference”, “Standard” and “SCCLim-I” is identical to the data shown in Sup. Fig. 15 – a and is repeated for comparability reasons. One-way ANOVA with Tukey´s multiple comparisons test was used. Statistical significance was defined as ** - p<0.01, **** - p<0.0001, ns – non significant (TIF 520 KB) [file 18_2022_4584_MOESM25_ESM.tif]

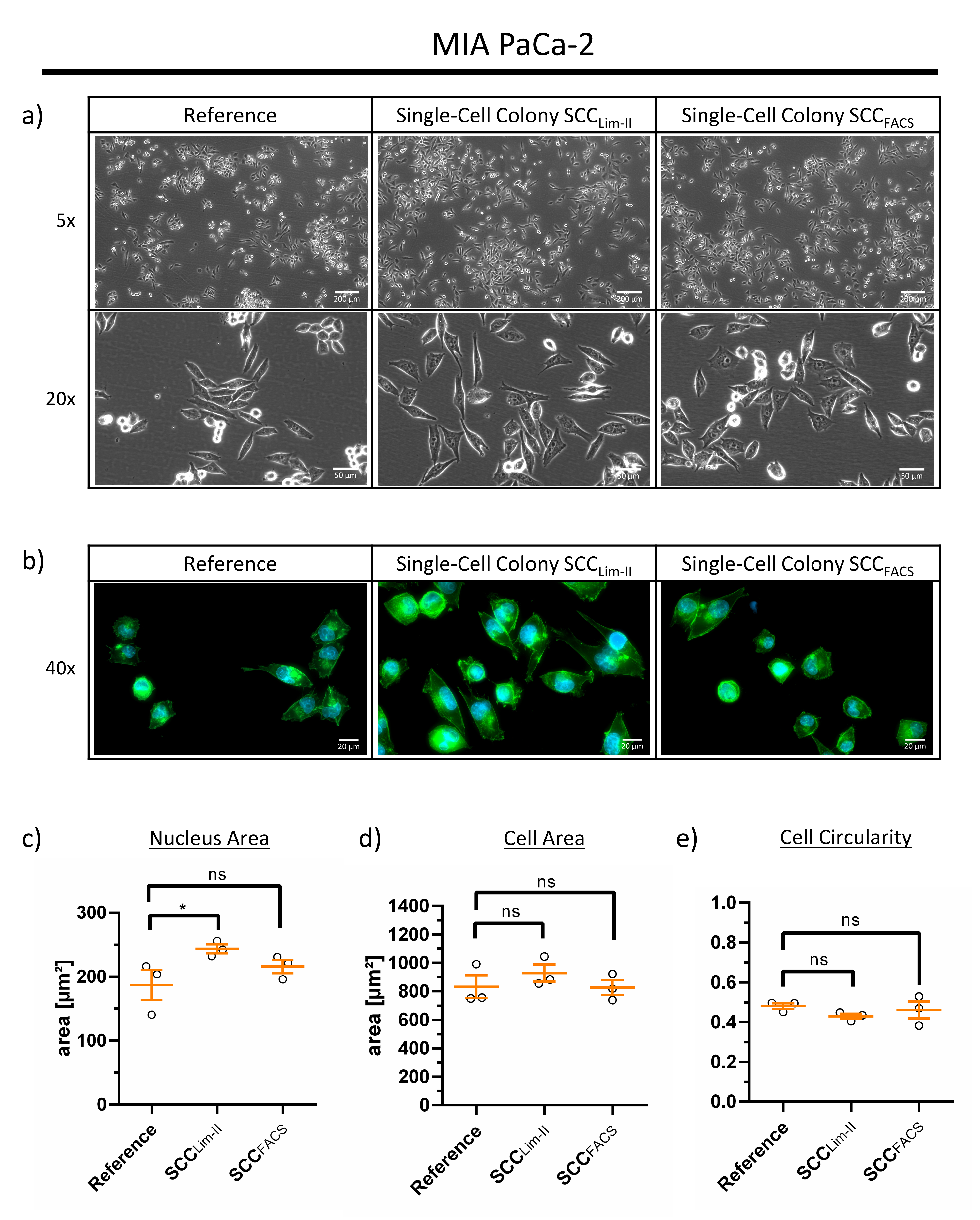

Supplement: Supplementary file 26 — Sup. Fig. 18 Cell Morphology before and after Clonal Isolation of human pancreatic MIA PaCa-2 cells via two rounds of limiting dilution or via FACS-assisted single-cell isolation. Human pancreatic MIA PaCa-2 reference cells either undergoing clonal isolation by two successive rounds of limiting dilution (SCCLim-II) or by one round of FACS-assisted single-cell isolation (SCCFACS). (a) Cells were cultured in cell culture flasks and imaged via phase contrast microscopy (5x and 20x magnification). (b) Cells were fluorescence stained for F-actin by fluorophore labelled Phalloidin (green) and dsDNA by Hoechst 33342 (blue). Maximum intensity projection of 40x z-stack images is shown. (c-e) Morphometric parameters of individual cells were analysed based on Phalloidin and Hoechst 33342 fluorescence staining. Three independent replicates and at least 216 nuclei (c) or 100 cells (d,e) per condition and replicate were analysed. Data for MIA PaCa-2 reference are identical to the data shown in Fig. 2 and is repeated for comparability reasons. Experimental and statistical analysis was performed together for all 5 MIA PaCa-2 cell lines. Scatter plot dots represent mean values per replicate (error bars show mean and S.E.M., * - p<0.05, ns – non significant) (TIF 7112 KB) [file 18_2022_4584_MOESM26_ESM.tif]

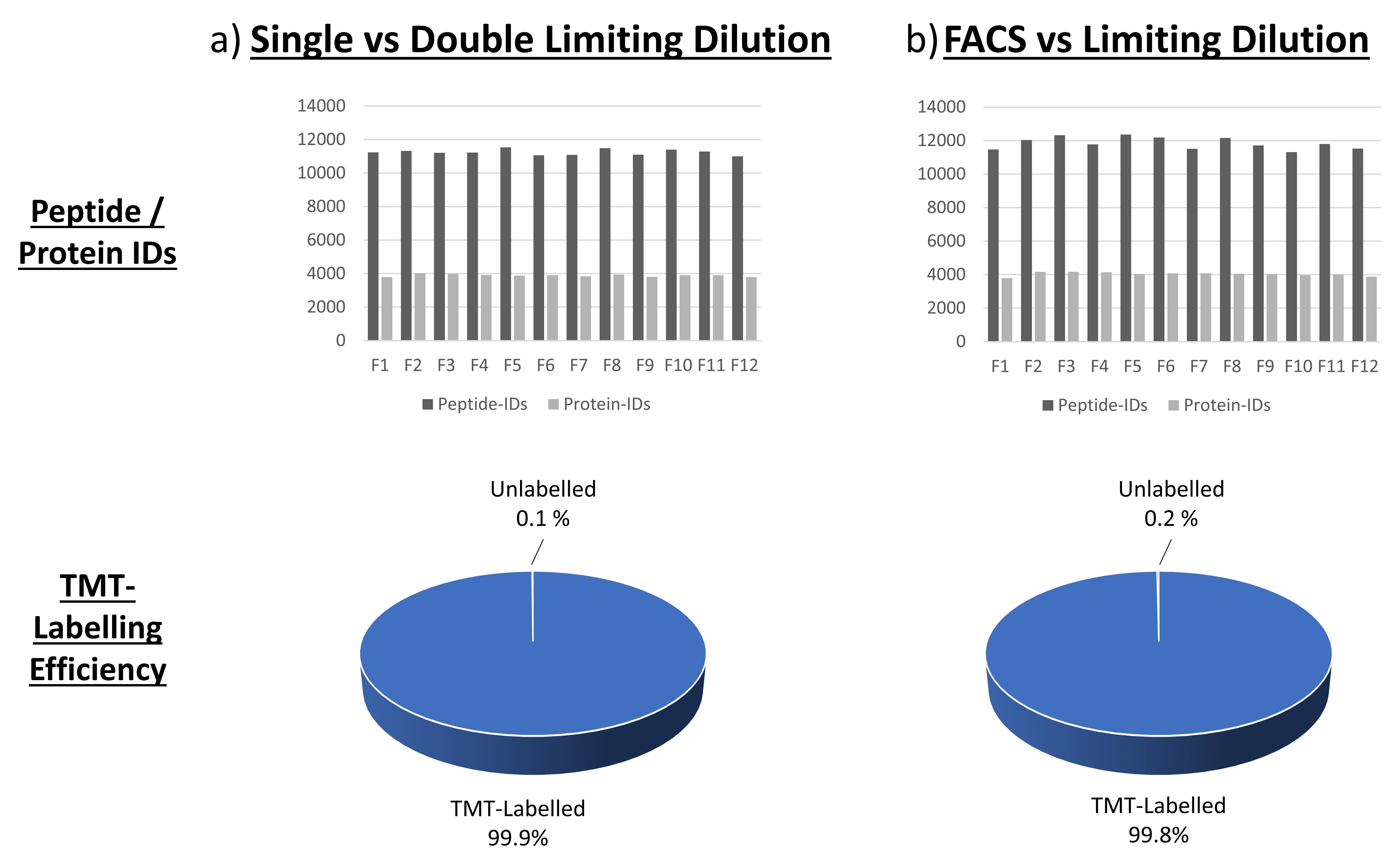

Supplement: Supplementary file 27 — Sup. Fig. 19 Proteomic Data Characteristics for datasets investigating repeated limiting dilution cloning and clonal isolation using FACS. Proteomes of human pancreatic MIA PaCa-2 reference cells were either compared to the proteome of (a) cells undergoing a single round or two successive rounds of limiting dilution or to the proteome of (b) cells undergoing a single round of clonal isolation but with different methods including limiting dilution or FACS. For peptide and protein identification, acquired LC-MS/MS data were searched against a human database using MaxQuant with 1 % false discovery rate (FDR). Resulting numbers of identified peptides and proteins of respective datasets are shown for each fraction (F1–F12) as well as the efficiency of peptide-level TMT-labelling, which was evaluated by calculating the intensity-ratio of N-terminally labelled, tryptic peptides against all identified tryptic peptides (TIF 796 KB) [file 18_2022_4584_MOESM27_ESM.tif]

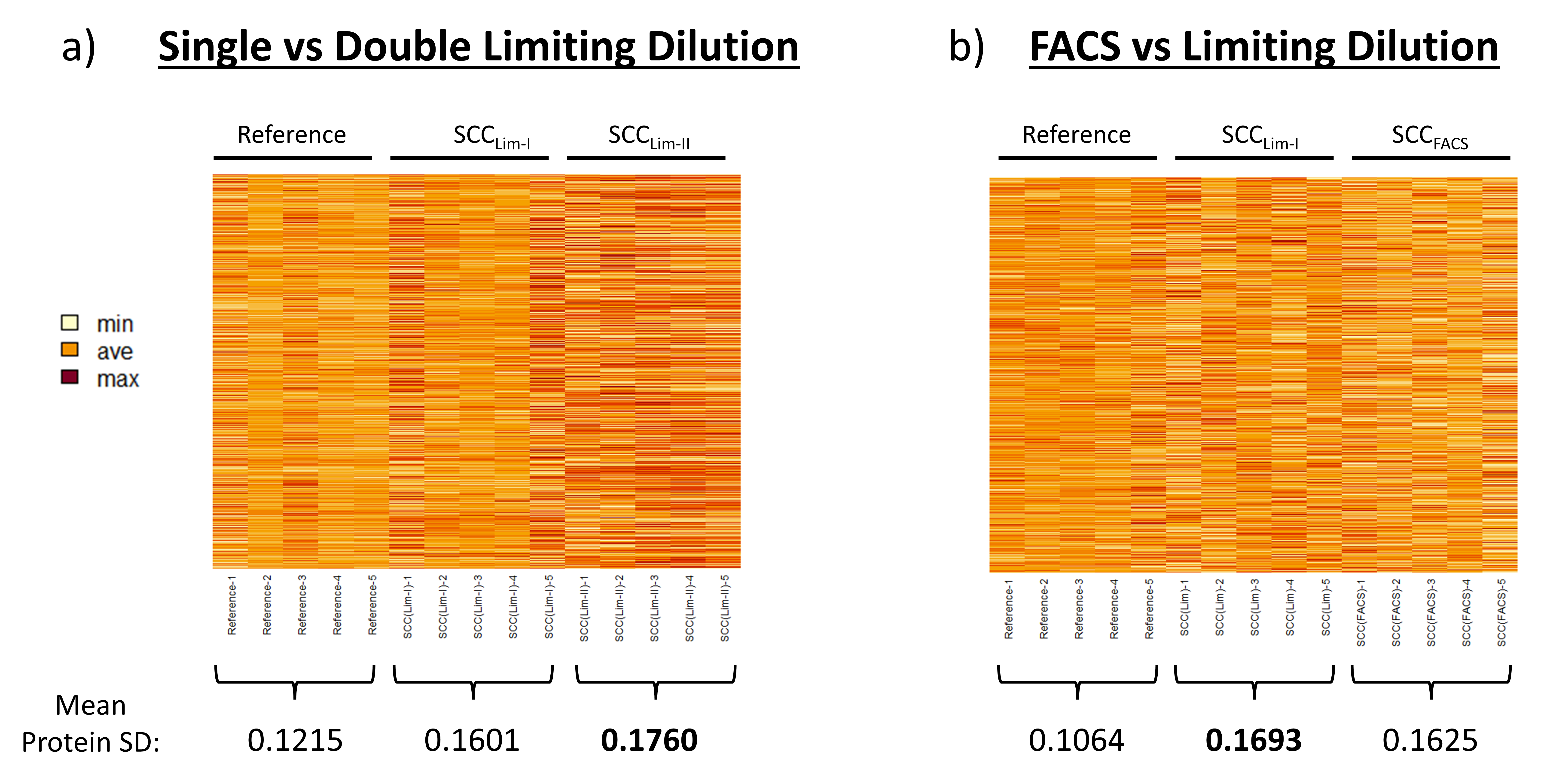

Supplement: Supplementary file 28 — Sup. Fig. 20 Heatmap Representation of Method Specific Protein Expression Profiles and Mean Protein Standard Deviation per Condition. Acquired proteomic datasets for MIA PaCa-2 either (a) undergoing several rounds of limiting dilution or (b) undergoing a single round of clonal isolation with different methods were visualised via heatmap representing proteins as rows, conditions (Reference, Single-cell colony SCC) as columns and the respective protein abundance as colour-coded field. Samples from similar conditions were grouped together and indicated accordingly above the heatmap. The mean protein standard deviation (SD) was calculated for each condition individually by determining the standard deviation of each protein across the 5 respective replicates before calculating the mean over all protein standard deviations. The highest mean protein standard deviation for each cell line is highlighted in bold (TIF 2438 KB) [file 18_2022_4584_MOESM28_ESM.tif]
